# Amyloid formation of alternatively spliced variants of α‐synuclein

**DOI:** 10.1002/pro.70195

**Published:** 2025-06-16

**Authors:** Daniel Q. SanGiovanni, Ryan P. McGlinchey, Jennifer C. Lee

**Affiliations:** ^1^ Laboratory of Protein Conformation and Dynamics, Biochemistry and Biophysics Center National Heart, Lung, and Blood Institute, National Institutes of Health Bethesda Maryland USA

**Keywords:** aggregation, alternative splicing, amyloid, circular dichroism, electron microscopy, fibril, kinetics, Parkinson's disease, α‐synuclein

## Abstract

Parkinson's disease, dementia with Lewy bodies, and multiple system atrophy are disorders characterized by the presence of cytosolic α‐synuclein (SNCA) amyloids. The gene *SNCA* is alternatively spliced, generating three variants of SNCA, missing exon 3 (SNCAΔ3) or 5 (SNCAΔ5), or both exons (SNCAΔ3Δ5). Despite purported upregulation in disease states, their pathological relevance is ill‐defined. Here, we investigated the amyloid formation of alternatively spliced variants under physiological conditions. Aggregation kinetics, secondary structure, and fibril morphology of N‐terminally acetylated SNCAΔ3, SNCAΔ5, and SNCAΔ3Δ5 were assessed by thioflavin‐T fluorescence, circular dichroism spectroscopy, and transmission electron microscopy, respectively. Compared to SNCA, both SNCAΔ5 and SNCAΔ3Δ5 aggregate faster and adopt a more twisted fibril morphology, whereas SNCAΔ3 is more sensitive to solution conditions, exhibiting similar or modestly faster aggregation kinetics compared to SNCA. Cross‐seeding experiments using spliced‐variant fibrils and soluble SNCA showed that despite fibril morphological differences, SNCAΔ5 were competent seeds for SNCA, which is explained by their similar protease‐K resistant regions. Contrastingly, neither SNCAΔ3 nor SNCAΔ3Δ5 fibrils cross‐seed SNCA, indicating exon 3 (residues 41–54) is essential in modulating fibril structure. Notably, SNCA aggregation is stimulated by sub‐stoichiometric amounts of soluble SNCAΔ5 and SNCAΔ3Δ5, but not SNCAΔ3, suggesting that exon 5 (residues 103–130) is more important in modulating aggregation kinetics. Taken together, we propose that alternatively spliced variants are pathogenic by exacerbating aggregation of the main SNCA isoform.

## 1 INTRODUCTION

Intracellular accumulation of α‐synuclein (SNCA) into amyloid fibrils is a common pathological hallmark of Parkinson's disease (PD), dementia with Lewy bodies (DLB), and multiple system atrophy (MSA), collectively known as synucleinopathies (Spillantini et al. 1998; Wakabayashi et al. 1998). Functionally, SNCA is thought to aid the exocytosis and regulation of synaptic vesicles (Burré et al. 2010; Burré et al. 2014; Kaur and Lee 2020). The protein is 140 amino acids in length, consisting of three regions: an amphipathic, lysine‐rich N‐terminus (residues 1–60), a hydrophobic non‐amyloid‐β component region (residues 61–95), and a highly negatively charged C‐terminus (residues 96–140). SNCA is conformationally labile; in solution, the protein is intrinsically disordered but adopts α‐helical structure upon lipid binding (Kaur and Lee 2021; Pfefferkorn et al. 2012) and a cross‐β fold upon self‐assembly into amyloid fibrils (Serpell et al. 2000). The canonical amyloid cores generally encompass residues 36–99 (Guerrero‐Ferreira et al. 2019; Li et al. 2018a; Li et al. 2018b; Ni et al. 2019; Tuttle et al. 2016). An amyloid‐structure to disease‐phenotype relationship has been recently established, where fibril structures determined for PD and DLB are conformationally distinct from those of MSA (Schweighauser et al. 2020; Yang et al. 2022).

**FIGURE 1:**
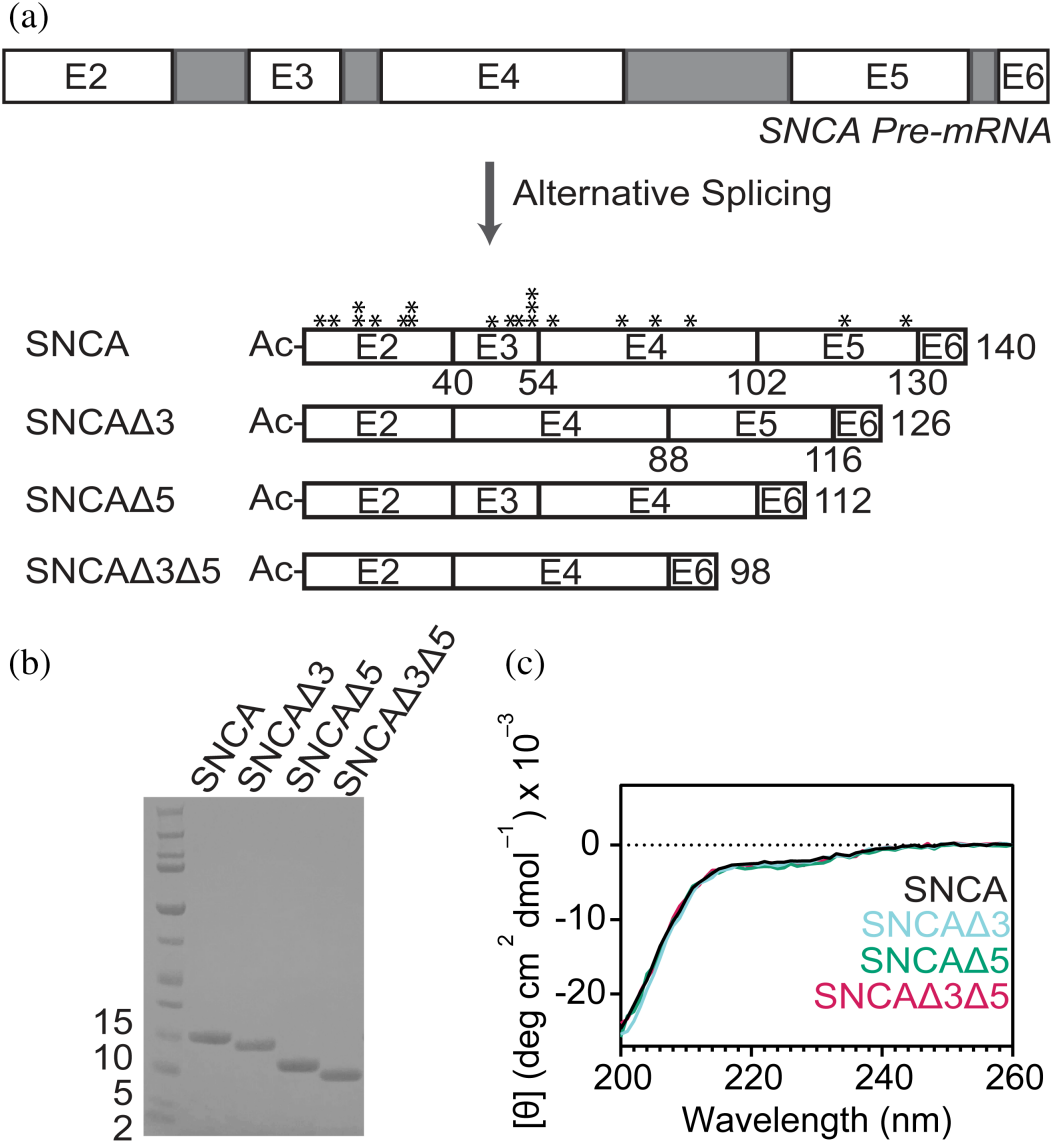
Characterization of N‐terminally acetylated (Ac) alternatively spliced variants of SNCA. (a) Schematic representation of the alternative splicing of the *SNCA pre‐mRNA* into SNCA and three distinct spliced variants via exon skipping. Exons and introns are colored white and gray, respectively. SNCAΔ3 and SNCAΔ5 lack either exon 3 or exon 5, whereas SNCAΔ3Δ5 lacks exons 3 and 5. Asterisk denotes known familial missense mutations (Fevga et al. 2021). Numbers denote the C‐terminal residue of the exon. (b) SDS‐PAGE (4–12% Bis‐Tris) analysis of purified recombinant full‐length SNCA and spliced variants. Corresponding LC–MS analysis is shown in Figure S1. (c) Comparison of averaged CD spectra of soluble SNCA and spliced variants (*n* = 3).

*SNCA* gene has been described to consist of six exons, where only exons 2–6 are found in the final translated protein, with exon 1 being an untranslated region (Bungeroth et al. 2014). However, it is important to mention that the current GenBank data show that *SNCA* contains 7 exons, with exons 3–7 being transcribed (NCBI accession number NG_011851). Nevertheless, we elected to use the former convention to ease comparison to the existing literature (Beyer and Ariza 2013). Although rare, examples of familial PD with SNCA missense mutations have been identified (Fevga et al. 2021) (Figure 1a) as well as *SNCA* duplication and triplication (Singleton et al. 2003; Singleton et al. 2004). Compared to wild‐type (WT) SNCA, many of these familial missense mutations such as E46K, H50Q, G51D, A53T/E, and E83Q accelerate aggregation under a variety of solution conditions (Buell et al. 2014; Flagmeier et al. 2016; Flynn et al. 2018; Kumar et al. 2022). Furthermore, fibril structures of E46K, H50Q, and G51D adopt distinct polymorphs compared to that of the WT protein (Boyer et al. 2019; Boyer et al. 2020; Sun et al. 2021; Zhao et al. 2020). These observations support a causal connection of both aggregation propensity and fibril structure to disease.

In addition to the main isoform, the *SNCA* gene generates three additional spliced variants through exon skipping (Beyer et al. 2008b; Campion et al. 1995; Ueda et al. 1994): SNCAΔ3 (missing exon 3, residues 41–54), SNCAΔ5 (missing exon 5, residues 103–130), and SNCAΔ3Δ5 (missing exons 3 and 5, residues 41–54 and 103–130) (Figure 1a). Interestingly, for PD patients, both mRNA levels of SNCAΔ5 and SNCAΔ3Δ5 are found to be upregulated in the cerebellum (Cardo et al. 2014), whereas for MSA patients only SNCAΔ5 mRNA is increased (Brudek et al. 2016). While literature reports changes in mRNA expression of spliced SNCA variants in disease (Beyer 2006; Beyer et al. 2004; Beyer et al. 2006; Beyer et al. 2008a; Beyer et al. 2008b; Beyer et al. 2009; Beyer and Ariza 2013; Brudek et al. 2016; Cardo et al. 2014), it is yet to be established whether these transcripts are translated into proteins. Nevertheless, since 95% of multi‐exon genes undergo alternative splicing to expand proteome diversity and function (Choi et al. 2023), it stands to reason that these SNCA proteoforms do have a biological function. Thus, it is imperative to understand the roles these variants play in synucleinopathies as an increased level of SNCA is associated with pathology.

Much of SNCA research has been devoted to understanding the aggregation behavior and fibril structure of full‐length SNCA because it may provide mechanistic insights into pathogenesis; however, to the best of our knowledge, there are only two aggregation studies on alternatively spliced SNCA variants. The first was in 2014 reporting on SNCAΔ3 and SNCAΔ3Δ5 (designated as SNCA‐126 and SNCA‐98) (Bungeroth et al. 2014). It was suggested that both variants aggregated to a lesser extent than full‐length based on a cellular assay, and only recombinant SNCA‐126 formed fibrillar material. Most recently, in vitro amyloid formation of all three isoforms was investigated in 2024 (Röntgen et al. 2024), and all three variants were shown to form fibrils. Interestingly, SNCAΔ5 (designated as SNCA‐112) was found to stimulate soluble SNCA aggregation through seeding and co‐mixing experiments (Röntgen et al. 2024).

It is important to mention that the two abovementioned studies did not use the physiologically relevant N‐terminally acetylated SNCA. In vivo, α‐syn is co‐translationally acetylated at the α‐amino N‐terminus (Anderson et al. 2006; Ohrfelt et al. 2011). This irreversible modification is common to eukaryotic proteins (Ree et al. 2018). N‐terminal acetylation of SNCA has been shown to influence both biological and pathological functions, enhancing lipid‐binding (Dikiy and Eliezer 2014; Maltsev et al. 2012; O'Leary et al. 2018) as well as slowing aggregation kinetics and affecting fibril polymorphism (Bell et al. 2022; Watson and Lee 2019). Furthermore, it was recently shown that N‐terminal acetylation impacted aggregation of five PD‐related SNCA variants (A30P, E46K, H50Q, G51D, and A53T) to varying degrees, highlighting the sensitivity of SNCA aggregation down to the smallest of molecular perturbations (Bell et al. 2023). Therefore, it is pertinent to further study N‐terminally acetylated forms of alternatively spliced SNCA variants.

Here, we examined the relative aggregation propensities of N‐terminally acetylated spliced variants of SNCA through aggregation kinetics, secondary structural characterization by circular dichroism spectroscopy, transmission electron microscopy, and limited protease digestion. Seeding and co‐mixing experiments are also performed to test the hypothesis that spliced variants can enhance the amyloid formation of the main SNCA isoform. Our data revealed under physiological salt conditions (140 mM NaCl) at cytosolic pH (pH 7.4), both SNCAΔ5 and SNCAΔ3Δ5 exhibit accelerated aggregation kinetics compared to that of SNCA, while SNCAΔ3 behaves similarly to SNCA. Importantly, SNCAΔ5 fibrils were competent seeds to template SNCA aggregation; moreover, SNCA aggregation was stimulated in the presence of substoichiometric amounts of soluble SNCAΔ5. In the case of SNCAΔ3Δ5, the protein also enhances SNCA aggregation upon co‐mixing, but for SNCAΔ3, its presence had no measurable effect on SNCA in either cross‐seeding or co‐mixing experiment. Collectively, this work suggests that SNCAΔ5 and SNCAΔ3Δ5 are more likely to contribute to the development of synucleinopathies.

## 2 RESULTS

### 2.1 Aggregation kinetics of alternatively spliced variants under high agitation condition

**FIGURE 2:**
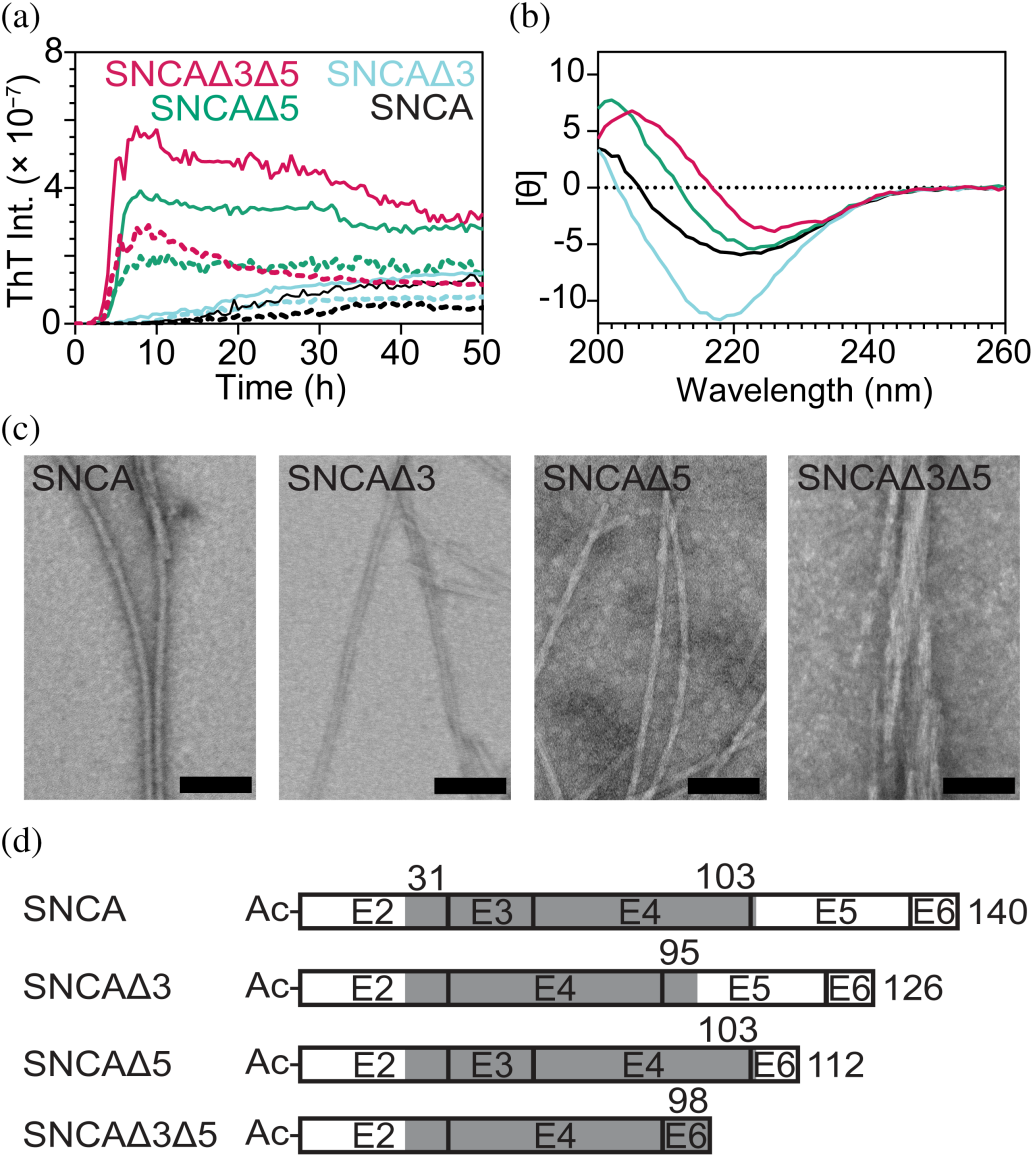
Amyloid formation of alternatively spliced variants under high agitation condition. (a) Comparison of aggregation kinetics monitored by ThT (20 mol%) fluorescence at two protein concentrations (average traces [*n* = 4] for 100 and 50 μM are shown as solid and dashed lines, respectively) in 20 mM NaPi, 140 mM NaCl, pH 7.4, shaken at 100 rpm and 37°C supplemented with a 2‐mm borosilicate bead. All kinetics data are shown in Figure S2. (b) Representative average CD spectra of insoluble fraction containing SNCA and spliced variant fibrils (*n* = 3) post‐aggregation. [θ] are in units of deg cm^2^ dmol^−1^ (× 10^−3^). Variants are color‐coded as in (a). (c) Representative TEM images taken of fibrillar SNCA and spliced variants aggregated between concentrations of 100–170 μM with beads. Scale bars are 100 nm. Larger fields of view are shown in Figure S3. (d) Smallest PK‐resistant cores of SNCA and spliced variants determined by LC–MS denoted by the gray box and indicated by the numbers (exact masses and representative SDS‐PAGE gels are shown in Table S1 and Figure S4, respectively).

N‐terminally acetylated SNCA and spliced variants (SNCAΔ3, SNCAΔ5, and SNCAΔ3Δ5) were purified to homogeneity via ion exchange chromatography (Figure 1b) and validated on liquid chromatography mass spectrometry (LC–MS; Figure S1, Supporting Information). Utilizing circular dichroism (CD) spectroscopy, all four SNCA isoforms were characterized to be intrinsically disordered in the soluble state (Figure 1c). To compare the aggregation propensities of the spliced variants to SNCA, a standard thioflavin T (ThT) fluorescence assay was conducted at two protein concentrations (50 and 100 μM, shaking in the presence of a single borosilicate bead) under physiological solution conditions (20 mM NaPi, 140 mM NaCl, pH 7.4 at 37°C). While nearly non‐emissive in buffer alone, ThT intensity increases upon binding to amyloid structure (Naiki et al. 1989). At both protein concentrations, aggregation kinetics of both SNCAΔ5 and SNCAΔ3Δ5 were substantially accelerated, exhibiting shorter lag times and growth phases, compared to those of SNCAΔ3 and SNCA (Figures 2a and S2). Under these solution conditions, SNCA and SNCAΔ3 display highly similar aggregation behaviors; though at 100 μM, SNCAΔ3 has a slightly reduced lag time.

### 2.2 Characterization of fibril secondary structure and morphology

Secondary structure and fibril morphology of the SNCA variants post‐aggregation was assessed using CD spectroscopy (Figure 2b) and transmission electron microscopy (TEM) (Figures 2c and S3), respectively. Unlike the soluble data, CD spectral differences are observed. The β‐sheet signature of SNCAΔ3 is more intense with a small, blue‐shifted wavelength maximum (218 nm) compared to that of SNCA at 220 nm. In contrast, the negative peaks of SNCAΔ5 and SNCAΔ3Δ5 red‐shift to longer wavelengths (~225 nm). This observation is similar to the red‐shifted peak (230 nm) reported for a C‐terminally truncated α‐synuclein variant, composed of residues 1–108 (Iyer et al. 2017), which is missing nearly all of exon 5 as well as exon 6. Interestingly, minimal secondary structural differences between the isoforms were observed by Fourier transform infrared spectroscopy (Röntgen et al. 2024). Filamentous aggregates are clearly visualized for all proteins by TEM, with SNCAΔ5 and SNCAΔ3Δ5 appearing more twisted than the 120 nm helical twist for SNCA (Ni et al. 2019) and SNCAΔ3 (Figures 2c and S3).

### 2.3 Determining PK‐resistant cores

Limited protease‐K (PK) digestion was performed to compare fibril stabilities towards proteolysis. Based on SDS‐PAGE analysis, SNCAΔ5 fibrils appear to be most resistant to PK degradation followed by SNCA, whereas both SNCAΔ3 and SNCAΔ3Δ5 are readily digested at high PK concentrations (Figure S4). The smallest PK‐resistant cores were determined by peptide mapping using LC–MS (Table S1): 31–103 (SNCA), 31–95 (SNCAΔ3), 31–103 (SNCAΔ5), and 31–98 (SNCAΔ3Δ5). While all four fibrils share the same N‐terminal cleavage at A30/G31 (where/denotes cut site), C‐terminal cleavages are more nuanced. For SNCA and SNCAΔ3, the same C‐terminal cleavage site was found at Q109/E110 (Q95/E96 in SNCAΔ3), yielding PK‐resistant cores composed of residues 31–109 and 31–95, respectively (Table S1). Although the cut site is preserved, the primary sequences differ due to the absence of exon 3 (Figure 2d). On the other hand, the SNCAΔ5 core (31–103) is the most conserved compared to that of SNCA, differing only by 1 residue (N103 for SNCA and E103 for SNCAΔ5). Notably, there were no C‐terminal PK cleavages for SNCAΔ3Δ5 fibrils resulting in a PK‐resistant fragment containing residues 31–98.

### 2.4 Aggregation kinetics of alternatively spliced variants under low agitation condition

**FIGURE 3:**
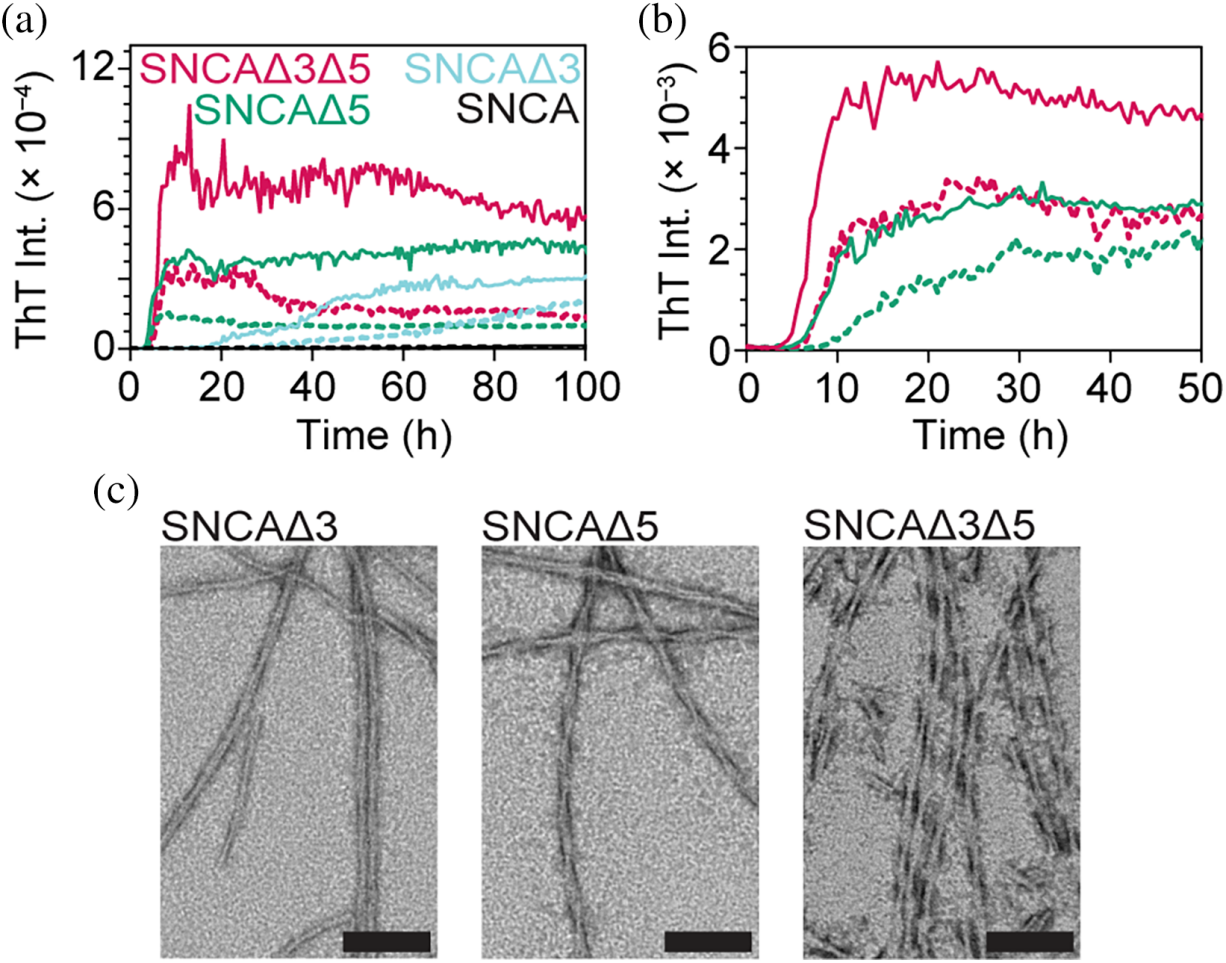
Amyloid formation of alternatively spliced variants under low agitation condition. (a) Comparison of aggregation kinetics monitored by ThT (20 mol%) fluorescence at two protein concentrations (average traces [*n* = 4] for 100 and 50 μM are shown as solid and dashed lines, respectively) in 20 mM NaPi, 140 mM NaCl, pH 7.4, shaken at 100 rpm and 37°C. All kinetics data are shown in Figure S5. (b) Comparison of SNCAΔ5 and SNCAΔ3Δ5 at lower protein concentrations (average traces [*n* ≥ 5] for 20 and 10 μM are shown as solid and dashed lines, respectively). Variants are color‐coded as in (a). All kinetics data are shown in Figure S6. (c) Representative TEM images of SNCA and spliced variants aggregated in the absence of beads. Scale bars are 100 nm. Larger fields of view are shown in Figure S7.

Because we were not able to differentiate between the two faster (SNCAΔ3Δ5 and SNCAΔ5) and between the two slower (SNCAΔ3 and SNCA) proteins, we adjusted the experimental conditions to reduce agitation by removing the borosilicate bead, which protracted aggregation kinetics (Figures 3a and S5). It is now resolved that SNCAΔ3 aggregates faster than SNCA, exhibiting the expected dependence on protein concentration, where at 100 μM, the lag time is markedly shortened compared to 50 μM. However, the kinetics of SNCAΔ3Δ5 and SNCAΔ5 remained indiscernible and unchanged from those collected in the presence of beads (Figures 2a and S2). Thus, we repeated the experiment at lower protein concentrations of 20 and 10 μM (Figures 3b and S6), which shows that SNCAΔ3Δ5 is the most aggregation‐prone of the four proteins. It is noted that the aggregation kinetics for SNCAΔ3Δ5 do not exhibit a strong protein concentration dependence between 10 and 100 μM. Using TEM, these fibril morphologies are reminiscent of those shown in Figure 2c, but with better staining (Figures 3c and S7), enabling quantitative determination of fibril half pitches for the two twisted fibrils (SNCAΔ5 = 75 ± 8 nm and SNCAΔ3Δ5 = 69 ± 9 nm; Figure S8). It is now apparent that SNCAΔ3 fibrils are heterogeneous, with populations of twisted as well as rod‐like fibrils. The formation of β‐sheets was confirmed by CD (Figure S9). As SNCA aggregation kinetics are accelerated under lysosomal solution conditions (Figure S10), we also evaluated the behavior of the variants at pH 5, where the relative aggregation trends hold.

### 2.5 SNCA aggregation is preferentially stimulated by SNCAΔ5 fibrils

**FIGURE 4:**
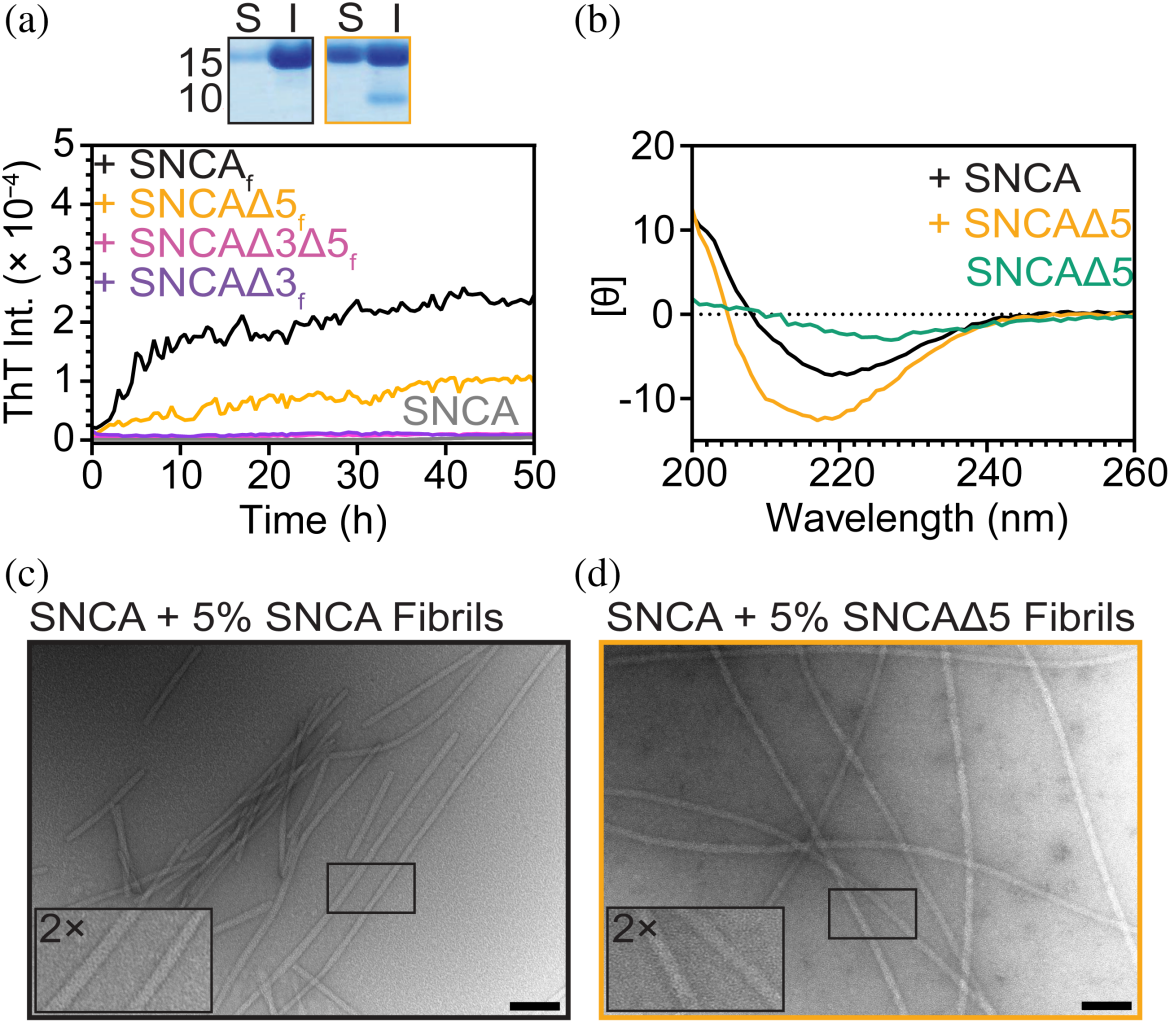
Cross‐seeding reactions of soluble SNCA with alternatively spliced variant fibrils. (a) Comparison of aggregation kinetics of SNCA (30 μM) in the presence of 1.5 μM preformed spliced variant fibrils (average of *n* = 5, in 20 mM NaPi, 140 mM NaCl, pH 7.4, [ThT] = 10 μM). See Figure S12 for all data. Data for 3 μM preformed spliced variant fibrils are shown in Figure S13. Subscript f denotes fibril. Top panels show SDS‐PAGE analysis of soluble (S) and insoluble (I) fractions from ultracentrifugation post‐aggregation of self‐seeded SNCA (left) and SNCA cross‐seeded with 5% SNCAΔ5 (right). (b) Representative CD spectra of insoluble fraction of SNCA seeded by SNCAΔ5. Spectra of self‐seeded and SNCAΔ5 fibrils alone are also shown for comparison (average of *n* = 3). [Θ] are in units of deg cm^2^ dmol^−1^ (×10^−3^). (c, d) Representative TEM image of self‐seeded SNCA and SNCA cross‐seeded with 5% SNCAΔ5 fibrils. Scale bars are 100 nm. Insets are 2× magnified views within the images.

After establishing the aggregation propensity of the spliced variants (SNCAΔ3Δ5 ≥ SNCAΔ5 >> SNCAΔ3 ≥ SNCA), we asked whether the spliced variant fibrils could cross‐propagate soluble SNCA under low agitation conditions. We first verified that all spliced variant fibrils had the ability to promote aggregation by self‐seeding, defined by the abrogation of the lag phase (Figure S11). Then, we tested the ability of each of the spliced isoforms to cross‐seed SNCA through the addition of 5 mol% of preformed fibrils (1.5 μM) to soluble SNCA (30 μM; Figures 4a and S12). Of the three spliced isoforms, only SNCAΔ5 fibrils were able to stimulate SNCA aggregation, albeit with a lower ThT intensity. To quantify fibril amounts, the resulting cross‐seeded SNCA fibrils were separated by ultracentrifugation and analyzed by SDS‐PAGE (Figure 4a, inset). The insoluble fraction contained ~60% protein, consistent with the lower ThT intensity. For SNCAΔ3 and SNCAΔ3Δ5 seeds, fibril propagation of SNCA did not occur, suggesting that both formed incompatible fibril polymorphs. However, upon doubling the seed concentration (10 mol%), some SNCA fibril formation was induced by SNCAΔ3 fibrils, potentially due to surface‐stimulated aggregation (Figure S13).

To determine whether fibril structure templating has faithfully occurred, we compared the secondary structure and fibril morphology of the cross‐propagated SNCA to that of the self‐seeded SNCA. Both cross‐ and self‐seeded SNCA exhibited similar CD spectra with a wavelength maximum at 220 nm, blue‐shifted from SNCAΔ5 seed alone (Figure 4b). TEM images taken post‐aggregation showed similar fibril morphologies for both self‐ (Figure 4c) and cross‐seeded samples (Figure 4d). Collectively, these results suggest that while the addition of SNCAΔ5 fibrils can stimulate SNCA aggregation by abolishing the lag phase, the twisted morphology of SNCAΔ5 fibrils is not propagated.

### 2.6 Aggregation of SNCA is stimulated by sub‐stoichiometric amounts of SNCAΔ5 and SNCAΔ3Δ5

**FIGURE 5:**
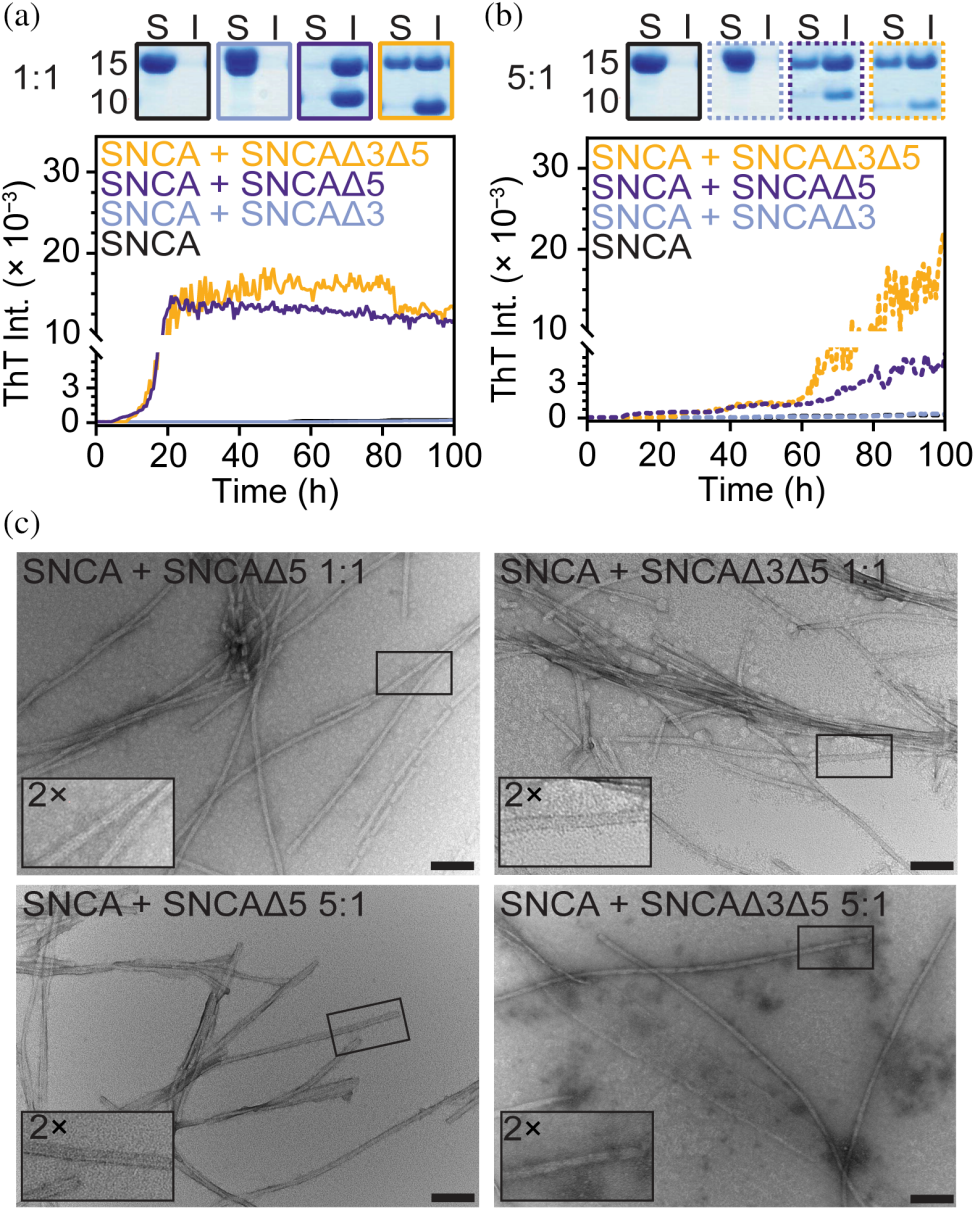
Co‐mixing reactions of soluble SNCA with alternatively spliced variant monomer. (a) Comparison of aggregation kinetics of SNCA (25 μM) in the presence of 25 μM spliced variant monomer (indicated as 1:1, an average of *n* ≥ 3) in 20 mM NaPi, 140 mM NaCl, pH 7.4, [ThT] = 5 μM. Top panels show SDS‐PAGE analysis of soluble (S) and insoluble (I) fractions after ultracentrifugation post‐aggregation of SNCA alone, SNCA co‐mixed with 25 μM SNCAΔ3, SNCAΔ5, and SNCAΔ3Δ5 (from left to right and colored as in the right panel). (b) Comparison of aggregation kinetics of SNCA (25 μM) in the presence of 5 μM spliced variant monomer (indicated as 5:1, average of *n* ≥ 3) in 20 mM NaPi, 140 mM NaCl, pH 7.4, [ThT] = 5 μM. See Figure S14 for all kinetics data. Top panels show SDS‐PAGE analysis of soluble (S) and insoluble (I) fractions after ultracentrifugation post‐aggregation of SNCA alone, SNCA co‐mixed with 5 μM SNCAΔ3, SNCAΔ5, and SNCAΔ3Δ5 (from left to right and colored as in the right panel). (c) Representative TEM images of SNCA co‐mixed with SNCAΔ5 (right panels) and SNCA co‐mixed with SNCAΔ3Δ5 in a 1:1 (top) and 5:1 (top) ratio. Scale bar are 100 nm. Insets are 2× magnified view within the images.

Next, we asked whether the soluble form of the spliced variants can affect SNCA aggregation directly. Aggregation reactions were performed under low agitation conditions with a fixed concentration of SNCA (25 μM) in the presence of either 25 or 5 μM of the spliced variant. Under these conditions, SNCA alone did not aggregate, simplifying interpretation. At an equimolar ratio (1:1), both SNCAΔ5 and SNCAΔ3Δ5 stimulated SNCA aggregation, whereas SNCAΔ3 showed no apparent change (Figures 5a and S14). SDS‐PAGE analysis is consistent, indicating increased protein levels relative to those of SNCA in the insoluble versus soluble fractions after ultracentrifugation. Although their ThT intensities are comparable, SDS‐PAGE results suggest that SNCAΔ5 is better at inducing SNCA aggregation, yielding more insoluble material. At sub‐stoichiometric concentrations (5:1 ratio), both SNCAΔ3Δ5 and SNCAΔ5 still stimulate SNCA, albeit at a slower rate (Figures 5b and S14). SDS‐PAGE analysis corroborates this observation by showing more than 50% of SNCA in the insoluble fractions. TEM images taken post‐aggregation of SNCA/SNCAΔ5 and SNCA/SNCAΔ3Δ5 co‐mixing revealed an eclectic mix of fibrils that were both rod‐like and twisted (Figure 5c).

## 3 DISCUSSION

The presence of alternatively spliced isoforms of SNCA is a natural event, yet their biological and pathological roles remain to be elucidated. That said, emerging evidence points to alternative splicing events that are also prevalent in other neurodegenerative disorders (Nikom and Zheng 2023), thereby strengthening the pathological implications of these SNCA isoforms. Here, we performed in vitro experiments characterizing amyloid formation of the three spliced variants of N‐terminally acetylated SNCA, missing residues 41–54 (SNCAΔ3) or 103–130 (SNCAΔ5), or both residues 41–54 and 103–130 (SNCAΔ3Δ5). All three isoforms aggregate faster than the full‐length protein (SNCAΔ3Δ5 ≥ SNCAΔ5 ≫ SNCAΔ3 ≥ SNCA), in agreement with a previous study on unacetylated proteins under solution conditions in the absence of NaCl (Röntgen et al. 2024), suggesting that neither N‐terminal acetylation nor NaCl has an impact on the relative aggregation propensity trend. A stimulatory effect on α‐synuclein aggregation by the removal of residues 103–130 is anticipated based on the fact that C‐terminal truncation 1–103 exhibits accelerated aggregation kinetics (Ni et al. 2019). However, the observation that the removal of residues 41–54 facilitated aggregation is surprising given that it has been reported that deleting residues 52–55 nearly abrogated amyloid formation (Shvadchak and Subramaniam 2014). That said, we speculate that the effect of exon 3 deletion is more like that of an N‐terminal truncation 41–140, which is more aggregation‐prone than SNCA (McGlinchey et al. 2021).

From a structural perspective, fibril distinctions were observed for the spliced variants by CD spectroscopy, PK digestion, and negative stain TEM, corroborating the importance of the two regions encoded by exons 3 and 5. Fibril morphology of variants with exon 5 removed was shown to be primarily twisted with a clearly defined helical pitch of 75 ± 8 and 69 ± 9 nm for SNCAΔ5 and SNCAΔ3Δ5, respectively (Figure 3c), which is highly reminiscent of the PD‐related C‐terminal truncation 1–103 (Ni et al. 2019). A structural similarity between the two spliced variants was also seen in the CD spectra (Figure 2b). On the other hand, limited PK digestion of SNCAΔ3Δ5 fibrils did reveal residues comprising exon 6 that are protease‐resistant, which were not observed upon digesting SNCAΔ5 fibrils (Figure 2d). This may infer that the additional C‐terminal residues that contribute to the SNCAΔ3Δ5 fibril core result in a distinct fibril structure.

For SNCAΔ3, fibrils were more heterogeneous with straight and twisted morphologies; variable helical pitches were also observed. This eclectic appearance suggests multiple distinct structures are present compared to SNCA fibrils, which are mostly rod‐like in appearance. Supporting this, PK digestion of SNCAΔ3 shows more PK resistance to SNCA in the C‐terminus by the absence of cutting at N89/E90 (N103/E104 in SNCA) (Table S1).

Using cross‐fibril propagation experiments, we conclusively show only SNCAΔ5 fibrils were capable of templating SNCA. This is noteworthy because the PD‐related C‐terminal truncation, where residues 103–140 are missing, is also shown to have a potent cross‐seeding effect (Ni et al. 2019; van der Wateren et al. 2018). In contrast, SNCAΔ3 and SNCAΔ3Δ5 fibrils, where residues 41–54 are absent, were incapable of promoting SNCA fibril formation, which is remarkably similar to the behaviors of ΔN‐terminal truncations (e.g., 36–140 and 41–140), which are poor seeds of the full‐length protein, suggesting a critical role for N‐terminal residues in SNCA propagation. Contrastingly, non‐acetylated variant fibrils were reported to enhance SNCA aggregation via cross‐seeding with varying efficacy: SNCAΔ5 > SNCAΔ3 > SNCAΔ3Δ5 (Röntgen et al. 2024), implicating an impact of N‐terminal acetylation on fibril polymorphism and compatibility, which has been documented for acetylated versus non‐acetylated SNCA (Watson and Lee 2019).

Our results can be explained by the inspection of the fibril structure of N‐terminally acetylated SNCA formed under similar conditions, which reveals residues 41–54 as part of the steric‐zipper interface (residues H50–E57) between the two protomers (Li et al. 2018a; Li et al. 2018b; Ni et al. 2019). Furthermore, there is an essential E46–K80 salt bridge in this structure, which would be abolished by removing residues 41–54 and explains why SNCAΔ3 and SNCAΔ3Δ5 form different fibril polymorphs. So, even when a template is provided, fibrils formed without exon 3 would be inaccessible to the full‐length protein. In light of this, the reported stimulation observed by SNCAΔ3 and SNCAΔ3Δ5 fibrils could likely represent surface‐enhanced aggregation, which at a higher seed concentration, we also observed (Figure S13).

The observation that SNCAΔ3 could not co‐aggregate with SNCA shows that exon 3 plays an essential role in establishing fibril polymorphism during primary nucleation. Interestingly, a double deletion mutant Δ36‐42 and Δ45‐57 of SNCA was shown not to aggregate, highlighting the importance of residues within and surrounding exon 3 (Doherty et al. 2020). Even though SNCAΔ3Δ5 could not cross‐seed, SNCAΔ3Δ5 was capable, along with SNCAΔ5 of stimulating SNCA through co‐aggregation (5:1; Figure 5b), suggesting a dominant effect of exon 5 removal. However, we note that this was not observed using a higher molar ratio (9:1) of SNCA to SNCAΔ3Δ5 (Röntgen et al. 2024). The observed stimulatory effect at sub‐stoichiometric protein concentration is biologically relevant because the expected population of the spliced forms would be substantially less than the full‐length. Mechanistically, based on the fact that both intra‐ and inter‐molecular electrostatic interactions between the N‐terminal (residues 1–60) and C‐terminal (residues 96–140) regions participate in early aggregation events (Bertoncini et al. 2005; Wu and Baum 2010), we propose that interactions of residues 103–130 are likely rate‐limiting for nucleation. Thus, aggregation is enhanced in the absence of exon 5. Collectively, this work offers mechanistic insights into the specific regions that are encoded by exons 3 and 5, delineating their roles in fibril formation with residues 41–54 modulating fibril structure and residues 103–130 regulating aggregation speed.

From a disease standpoint, our results can rationalize the consequences of having increased levels of SNCAΔ5 in MSA (Brudek et al. 2016), DLB (Beyer et al. 2004; Beyer et al. 2008a) and SNCAΔ5 and SNCAΔ3Δ5 in PD (Cardo et al. 2014) based on their increased aggregation propensity. This is particularly true for the upregulation of SNCAΔ5 in MSA, which is considered the most aggressive form of synucleinopathies. On the other hand, because SNCAΔ3 has no effect on full‐length SNCA, this variant is considered to be more benign. Similarly, based on its reduced mRNA levels in DLB (Beyer et al. 2006), it has been suggested that SNCAΔ3 could have a potential protective role as an aggregation‐inhibiting isoform. However, the fact that it forms a distinct fibril conformer, SNCAΔ3 could still exert an impact on disease etiology since there is a strong link between fibril structure and disease phenotype. While these results are promising, it is pertinent for future work to determine the extent of SNCA spliced isoforms at the protein level in patient‐derived tissues, requiring selective antibodies and sensitive proteomic approaches in order to address their importance in pathogenesis.

## 4 MATERIALS AND METHODS

### 4.1 Reagents

All reagents were obtained from Sigma unless otherwise noted.

### 4.2 Protein expression and purification

Plasmids pT‐7 (Paleologou et al. 2008) for SNCA and pET21a(+) for the three spliced variants (missing exon 3, exon 5, and exons 3 and 5 were purchased from GenScript) contained a silent mutation (TAT) at residue 136 to avoid the spontaneous mutation of Tyr‐to‐Cys (Masuda et al. 2006). Constructs were verified by DNA sequencing (Psomagen, USA). N‐terminally acetylated proteins were expressed in *E. coli* BL21(DE3) (New England Biolabs) by co‐transforming with yeast NatB genes (Johnson et al. 2010) as previously described (Watson and Lee 2019). Cells were induced with 1 mM IPTG once an OD of 0.8 was reached for 3 h. Cells were collected by centrifugation (6000 rpm at 4°C for 30 min) using a Sorvall SLC‐6000 rotor. Cell pellets were stored at −25°C until use.

For lysis, the previously published protocol was followed with some modifications for the spliced variants (Watson and Lee 2019). Briefly, the cell pellet (7 g) was first resuspended in 70 mL lysis buffer (100 mM Tris, 300 mM NaCl, 1 mM EDTA at pH 8.0 containing 1 mM PMSF) and sonicated with ½″ tip (Branson Sonifier, Duty Cycle 50%, Output Level 5) on ice for 15 min. The lysate was heat treated in a boiling water bath for 20 min and spun down at 18,000 rpm at 4°C for 30 min (Sorval SS34). Supernatant was then titrated to pH 3.5 before centrifugation at 18,000 rpm at 4°C for 30 min (Sorval SS34). All proteins were acidified except SNCAΔ5. While SNCAΔ5 was dialyzed in 20 mM MES, 1 mM EDTA, 0.5 mM PMSF, pH 6.0 overnight at 4°C, all the other three were dialyzed in 20 mM Tris, 1 mM EDTA, 0.5 mM PMSF, pH 8.0 before ion exchange chromatography using the ÄKTA Pure Protein Purification System (GE Healthcare).

SNCA and SNCAΔ3 cell lysates were applied to HiPrep DEAE 16/10 (GE Healthcare), followed by purification on Mono Q 16/10 (Pharmacia) column using 20 mM Tris, pH 8, and a linear NaCl gradient. SNCA and SNCAΔ3 eluted at ~240 and ~230 mM NaCl, respectively. The protocols for SNCAΔ3Δ5 and SNCAΔ5 differ. SNCAΔ3Δ5 cell lysate was first applied to HiPrep Q 16/10 (GE Healthcare), followed by Mono Q 16/10 (Pharmacia) using 20 mM Tris, pH 8, and a linear NaCl gradient. SNCAΔ3Δ5 eluted at ~80 mM NaCl. SNCAΔ5 cell lysate was applied to HiPrep SP 16/10 (GE Healthcare), followed by Mono S 10/10 (GE Healthcare) using 20 mM MES, pH 6 buffer, and eluted with a linear NaCl gradient. SNCAΔ5 eluted at ~60 mM NaCl. Fractions were selected and pooled based on protein purity assessment via SDS–PAGE analysis and UV absorption spectroscopy (CARY 300, Agilent). Pooled fractions were concentrated using an Amicon stirred cell (YM3 membrane) to typically approximately 170 μM, flash frozen with liquid nitrogen, and stored at −80°C. Purity and identity were verified by LC–MS (NHLBI Biochemistry Core). Using Agilent Mass Hunter software, SNCA, SNCAΔ3, SNCAΔ5, and SNCAΔ3Δ5 were seen to have >99% acetylation (Figure S1).

### 4.3 Circular dichroism spectroscopy

CD spectra were collected in 1‐mm quartz cuvettes using a Jasco J‐715 spectropolarimeter (Jasco Analytical Instruments) with the following settings: 200–260 nm, continuous mode, 100 nm min^−1^ scan rate, 1 nm bandwidth, 1 nm steps, and three accumulations at 20°C. Data are reported as the mean residue ellipticity [Θ] to account for differences in peptide length using the equation: [Θ] = (100θ)/(*cln*), where *θ* is the measured CD signal in mdeg, *c* is the concentration of the protein in mM, *l* is the path length of the cuvette in cm, and *n* is the number of residues in the protein. The formula for mean residue ellipticity was obtained from a previous paper (McGlinchey et al. 2014).

### 4.4 Aggregation kinetics

Using Zeba Spin Desalting Columns (Themo Scientific), proteins were desalted into aggregation buffer (20 mM NaPi, 140 mM NaCl, pH 7.4). Proteins were then filtered through ultra‐centrifugal filter tubes (100 kDa MWCO, Millipore Sigma). Protein concentrations were determined via UV spectroscopy using the absorbance at 280 nm with the molar extinction coefficient of 5960 M^−1^ cm^−1^ for SNCA and SNCAΔ3 and with the molar extinction coefficient of 4470 M^−1^ cm^−1^ for SNCAΔ5 and SNCAΔ3Δ5; these values are based on tyrosine content (1490 M^−1^ cm^−1^). Protein solutions were then diluted to their specified concentration using additional aggregation buffer. Sodium azide (1%) was added to each condition to achieve a final concentration of 0.02%. ThT was added to achieve a final concentration of 20 mol%. Solutions (70 μL) were then added to a 384‐well black, polypropylene, flat‐bottom microplate (781,209, Greiner Bio‐one) in replicates in the absence or presence of a 2‐mm borosilicate bead (described otherwise as low or high agitation conditions, respectively), sealed with an adhesive film, and placed in the Clairo Star^Plus^ microplate reader (BMG Lab Tech) equilibrated at 37°C. Plates were shaken using a single orbital pattern at 100 rpm, and ThT emission was collected at 30‐min intervals (excited at 415 nm (20 lamp flashes) and emission collected at 480 nm). At least two independent experiments using multiple biological replicates were conducted for each experimental condition. We note that while well‐to‐well and plate‐to‐plate variations were seen, especially for the low agitation condition, the relative trends were consistent and reproducible.

### 4.5 Transmission electron microscopy

Protein samples from high agitation conditions (10 μL of 12.5–25 μM) were added to TEM grids (copper coated and 400‐mesh formvar, Electron Microscopy Sciences) and incubated for 2 min. Grids then were wicked with filter paper, washed twice with 10 μL Milli‐Q water, followed by 10 μL of 1% (w/v) uranyl acetate, and incubated for 2 min before wicking away the liquid with filter paper. Grids were analyzed using JEOL JEM 1400 transmission electron microscope (accelerating voltage 120 keV) equipped with an AMT NANOSPRT43 digital camera with a MRK2‐Lens. For low‐agitation conditions, protein samples (10 μL of 16–33 μM) were added to the TEM grid following the sample protocol above. Protein samples from low agitation condition grids were analyzed using JEOL JEM 1200EX transmission electron microscope (accelerating voltage 80 keV) equipped with an AMT XR‐60 digital camera (NHLBI EM Core Facility). Fibril helical twists were calculated using the image processing package, ImageJ.

### 4.6 Limited proteinase‐K digestion

Proteinase K (Invitrogen) was added (20 μg mL^−1^) to each protein sample (30 μM) in a total reaction volume of 50 μL in 20 mM NaPi, 140 mM NaCl, pH 7.4. Reactions were incubated overnight in the incubating microplate shaker (VWR) at 600 rpm and 37°C. Reactions were terminated with 6M guanidinium hydrochloride and visualized on SDS–PAGE gels (Invitrogen) stained with Simply Blue. Proteolyzed samples (5 μL) supplemented with 0.1% TFA were injected into a reverse phase HPLC (High‐performance liquid chromatography) (Agilent 1290 series UHPLC, Agilent Technologies) with a Zorbax 300SB‐C18 (2.1 × 50 mm, 3.5 μm, Agilent Technologies) and introduced into the mass spectrometer as described (Apffel et al. 1995; Taggart et al. 2000). The initial condition of mobile phase is 100% of solvent A (0.05% trifluoracetic acid in Milli Q water), and proteins were eluted by a linear gradient of 2% per min of solvent B (0.05% trifluoroacetic acid in acetonitrile) with a flow rate of 200 μL per min. The protein effluent from the column was mixed in a tee with 100 μL per min glacial acetic acid just prior to the ion source of mass spectrometer to displace the protein bound trifluoroacetic acid (Taggart et al. 2000). Positive ion electrospray ionization (ESI) mass spectra for intact protein were obtained with an Agilent 6230 mass spectrometer equipped with a Dual ESI interface and a time‐of‐flight (TOF) mass detector (Agilent Technologies). Mass spectra were analyzed and deconvoluted using MassHunter Qualitative Analysis ver. B.07.00 software (Agilent Technologies). Using Agilent Mass Hunter software and the FindPept software (Expasy), the smallest fibrillar protease‐resistant core was determined for each of the proteins. Theoretical masses of fragments were determined using ProtParam software (Expasy). Results are shown in Table S1.

### 4.7 Fibril formation for seeding experiments

Fibrils were formed similarly as described above in the presence of a 2‐mm borosilicate bead at 37°C for 4–6 days, except ThT was omitted. Fibrils were spun down at 100,000*g* in an ultra‐centrifuge (Beckman Coulter Optima™ Max‐XP Ultracentrifuge) for 30 min at 19°C. Soluble fraction was removed. The pelleted fraction containing fibrils was then resuspended in equal volume of aggregation buffer. Fibril concentration was determined after denaturing in 4M guanidinium hydrochloride for 30 min by absorbance measurements using their respective molar extinction coefficients at 280 nm. Seeding conditions were set up using the protocol above, but the experimental conditions were 30 μM monomeric solution with 5 and 10% seeds in the absence of beads.

## AUTHOR CONTRIBUTIONS

**Daniel Q. SanGiovanni:** Conceptualization; writing – original draft; formal analysis; data curation. **Ryan P. McGlinchey:** Conceptualization; writing – original draft; data curation; formal analysis. **Jennifer C. Lee:** Conceptualization; writing – review and editing; supervision.

## CONFLICT OF INTEREST STATEMENT

The authors declare no conflicts of interest.

## Supporting information


**Data S1.** Supporting Information.

## Data Availability

The data that support the findings of this study are openly available in Figshare at https://doi.org/10.25444/nhlbi.28930919.
