# Supplementary material for: Amyloid formation of alternatively spliced variants of α‐synuclein
Source: Protein Sci. 2025 Jun 16;34(7):e70195. doi: 10.1002/pro.70195 (PMC12168488; doi:10.1002/pro.70195)
Supplement: Supplementary file 1 — Data S1. Supporting Information. [file PRO-34-e70195-s001.pdf]

Supporting Information

for

Amyloid formation of alternatively spliced variants of α-synuclein

Daniel Q. SanGiovanni, Ryan P. McGlinchey, and Jennifer C. Lee*

Table S1. MS analysis of limited PK-digestion of fibrils

Fig. S1. LC traces of purified SNCA and spliced variants

Fig. S2. Aggregation kinetics of SNCA and spliced variants under high agitation conditions

Fig. S3. Full TEM images of SNCA and spliced variants under high agitation conditions

Fig. S4. SDS-PAGE analysis of PK-digestion of fibrils

Fig.S5. Aggregation kinetics of SNCA and spliced variants under low agitation conditions

Fig.S6. Aggregation kinetics of SNCAΔ5 and SNCAΔ3Δ5 at low protein concentrations

Fig. S7. Full TEM images of SNCA and spliced variants under low agitation conditions

Fig. S8. Histogram reporting fibril half pitches of SNCAΔ5 and SNCAΔ3Δ5

Fig. S9. CD spectra of SNCA and spliced variants post-aggregation under low agitation conditions

Fig. S10. Comparison of aggregation kinetics of SNCA and spliced variants at pH 5

Fig. S11. Self-seeding reactions of SNCA and spliced variants at seed concentration of 5 mol%

Fig. S12. Cross-seeding reactions of soluble SNCA with spliced variant fibrils at a seed concentration of 5 mol%

Fig. S13. Cross-seeding reactions of soluble SNCA with spliced variant fibrils at a seed concentration of 10 mol%

Fig. S14. Co-mixing reactions of soluble SNCA with alternatively spliced variant monomer

Table S1. MS analysis of N-terminally acetylated SNCA and isoform variant fibrils (30 μM) incubated with PK (20 μg/mL) for 16–18 h at pH 7.4 and 37 °C.

SNCA Fibrils (30 μM) + 20 μg/mL protease K

Experimental Mass (Da)	Theoretical Mass (Da)	Sequence of Fragment
14502.65	14502.16	1–140
10957.80	10957.47	1–109
8280.60	8280.38	31–113
7868.14	7867.89	31–109
7256.47	7256.28	31–103

SNCAΔ3 Fibrils (30 μM) + 20 μg/mL protease K

Experimental Mass (Da)	Theoretical Mass (Da)	Sequence of Fragment
11395.33	11394.85	1–111
10018.75	10018.43	1–99
9606.41	9605.15	1–95
8305.58	8305.27	31–111
6929.08	6928.85	31–99
6516.70	6516.37	31-95

SNCAΔ5 Fibrils (30 μM) + 20 μg/mL protease K

Experimental Mass (Da)	Theoretical Mass (Da)	Sequence of Fragment
11414.35	11413.92	1–112
10879.59	10879.33	5–112
10450.00	10449.76	9–112
8324.55	8324.35	31–112
7271.58	7271.30	31–103

SNCAΔ3Δ5 Fibrils (30 μM) + 20 μg/mL protease K

Experimental Mass (Da)	Theoretical Mass (Da)	Sequence of Fragment
10062.70	10062.40	1–98
8157.24	8157.14	19–98
7115.21	7114.98	29–98
6973.06	6972.92	31–98

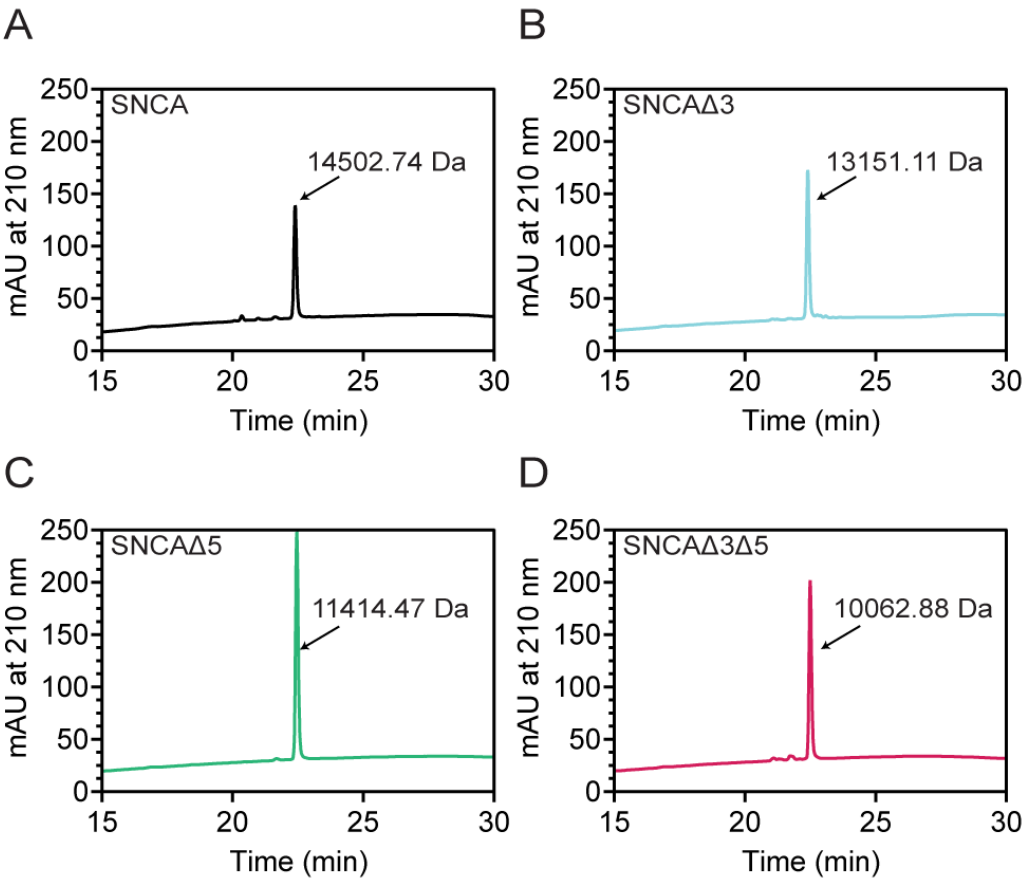

Figure S1. LC traces of acetylated SNCA (**A**) and spliced variants of SNCA (**B**-**D**) after purification. Experimental masses are shown.

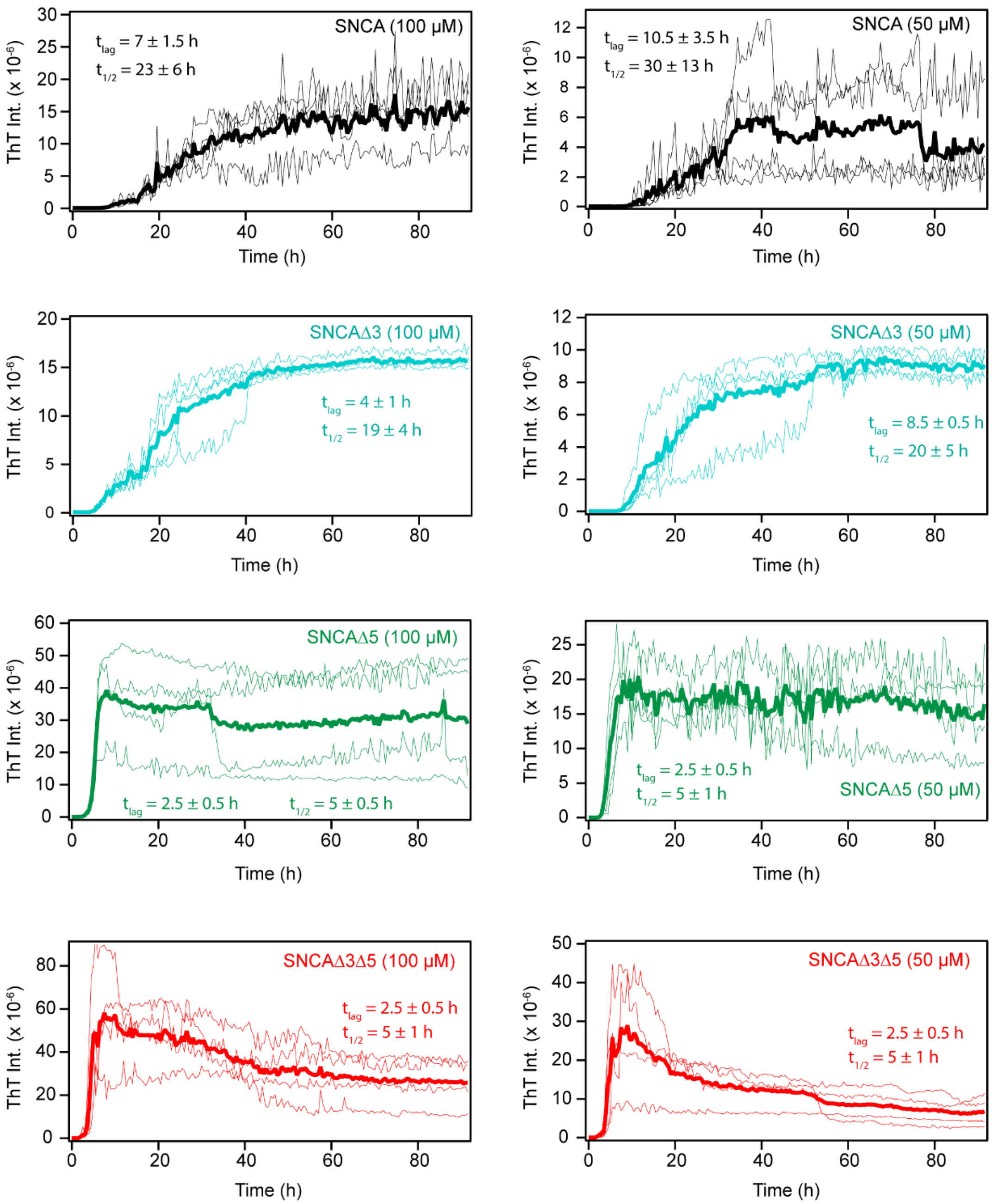

Figure S2. Amyloid formation under high agitation conditions. Aggregation kinetics monitored by ThT (20 mol%) fluorescence (n = 4) at two protein concentrations (100 and 50 μM) in 20 mM NaPi, 140 mM NaCl, pH 7.4, shaken at 100 rpm and 37 °C supplemented with a 2-mm borosilicate bead. Averaged curves shown in **Fig. 2A** are shown as solid lines. Mean and SD values for t_{lag} and $t_{½}$ are reported.

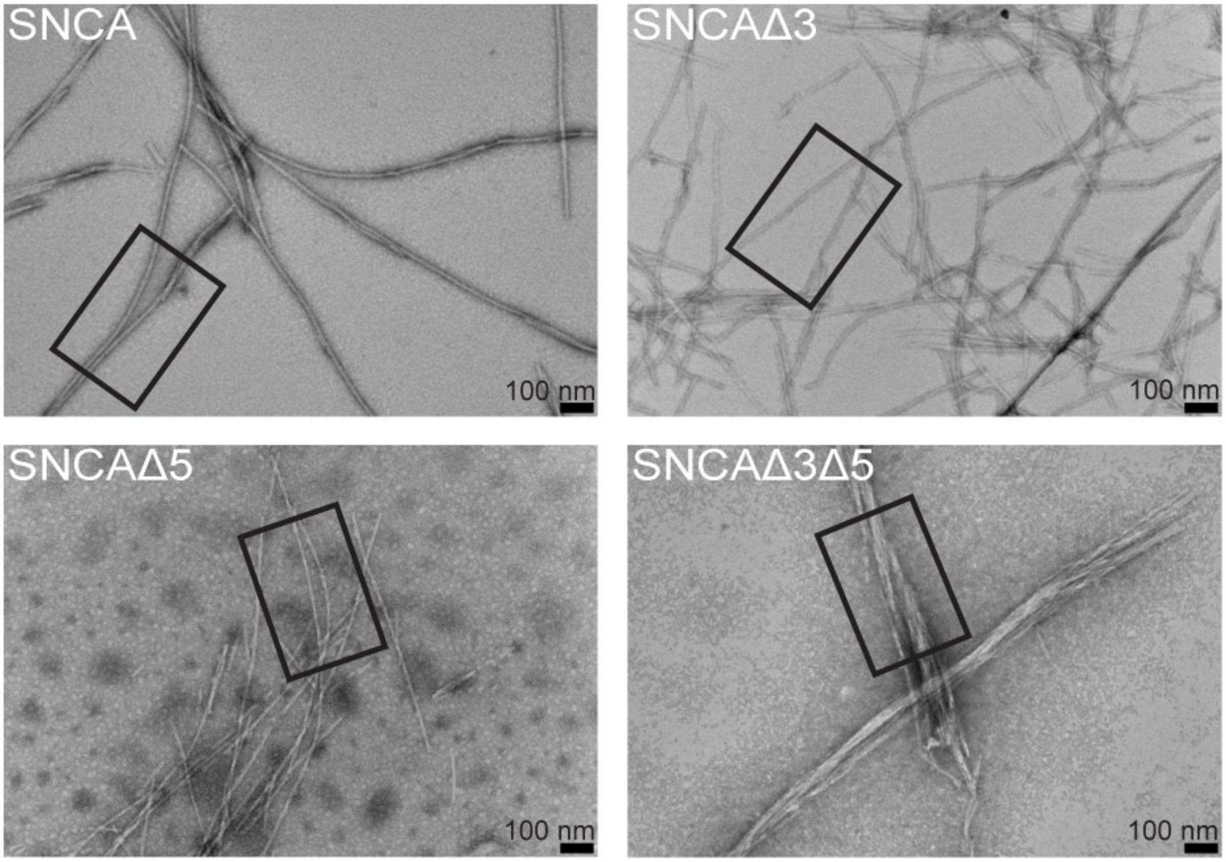

Figure S3. Full TEM images of fibrillar SNCA and spliced variants aggregated in the presence of beads. Fibrils were formed at 100–170 µM. Black box corresponds to field of view used in **Fig. 2C**.

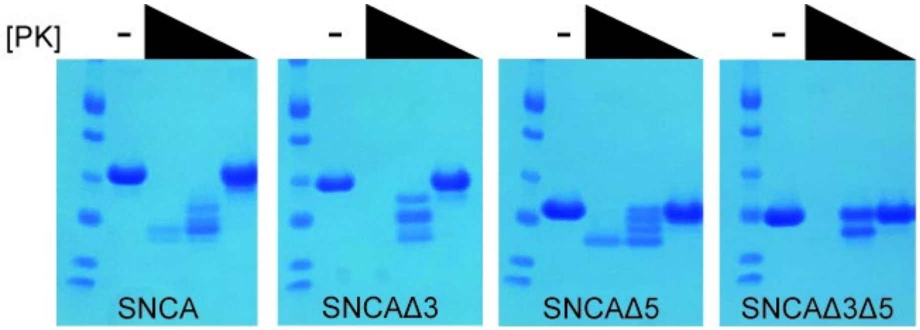

Figure S4. SDS-PAGE analysis of PK digestion of fibrils formed by SNCA and spliced variants (30 μM) as a function of PK concentration. Higher PK at 20 μg/mL (second lane from the right) was used for MS analysis shown in Table S1.

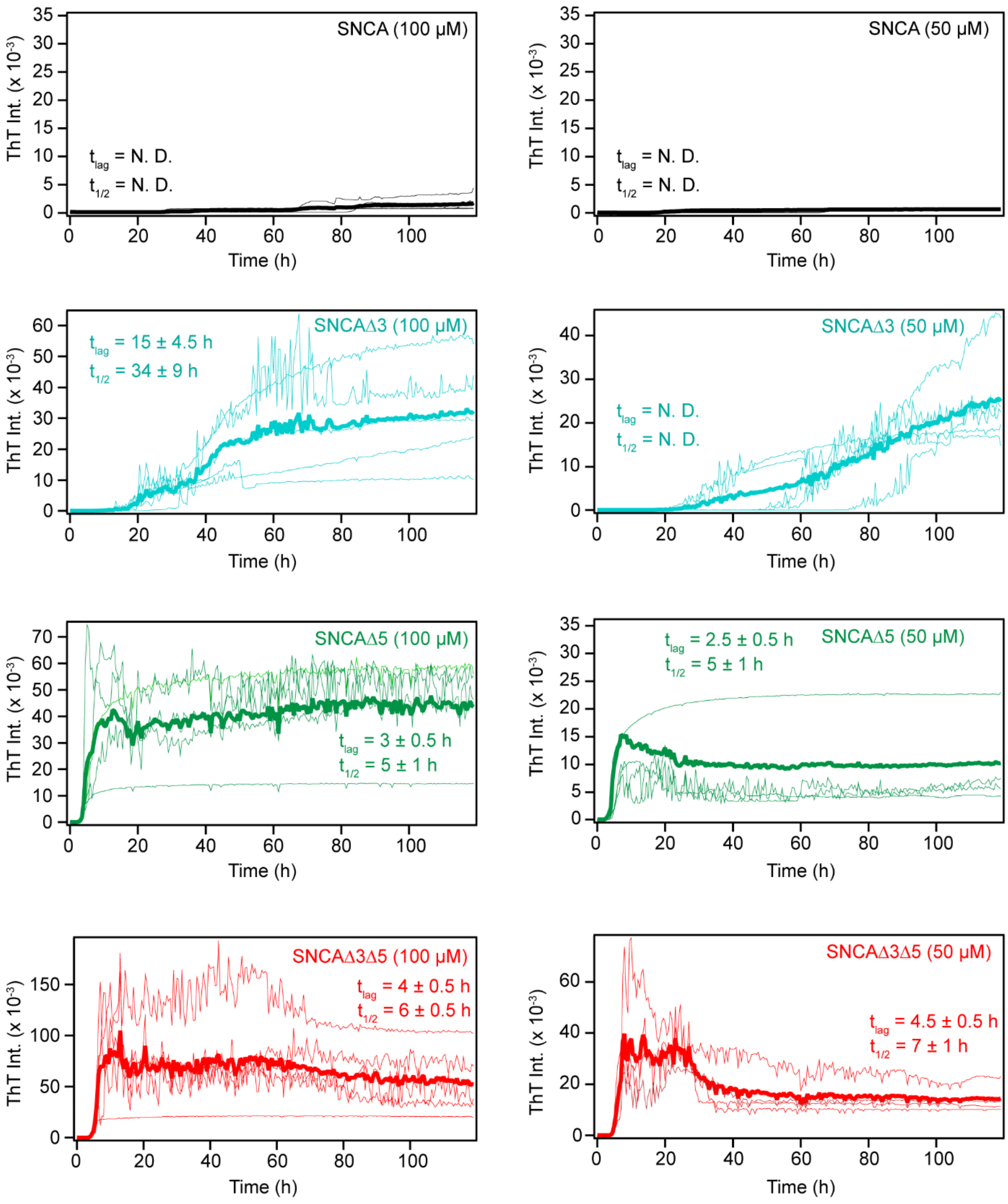

Figure S5. Amyloid formation under low agitation conditions. Aggregation kinetics monitored by ThT (20 mol%) fluorescence (n = 4) at two protein concentrations in 20 mM NaPi, 140 mM NaCl, pH 7.4, shaken at 100 rpm and 37 °C. Averaged curves shown in **Fig. 3A** are shown as solid lines. Mean and SD values for t_{lag} and $t_{½}$ are reported.

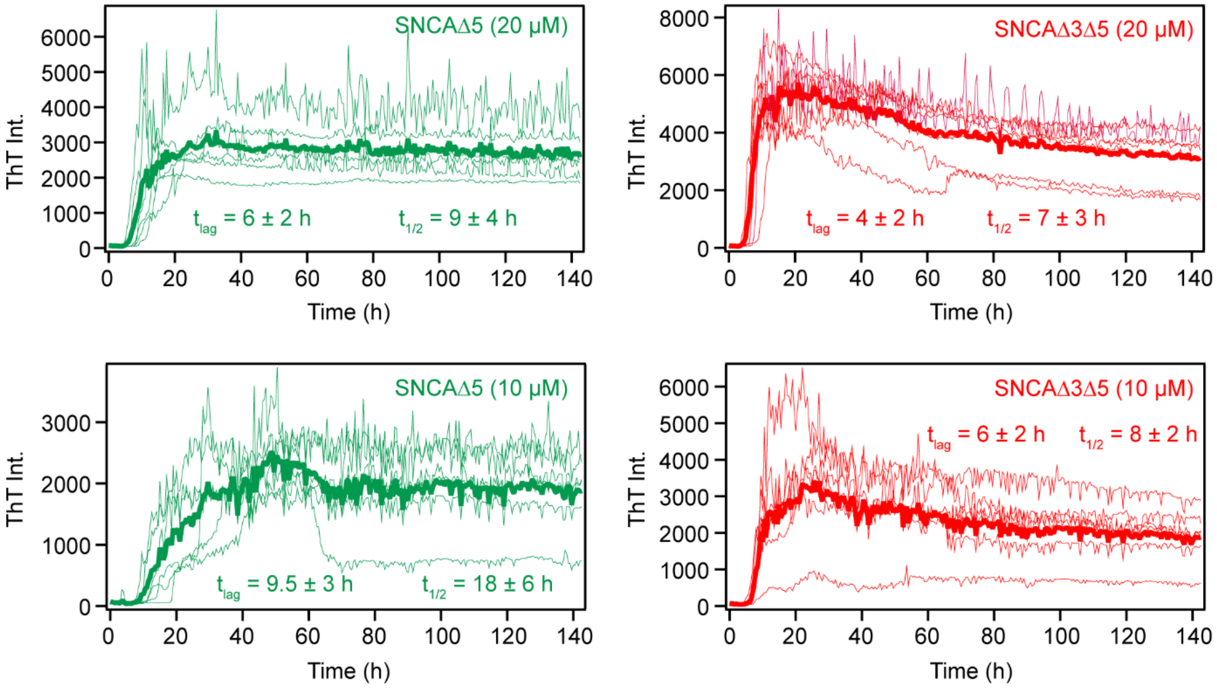

Figure S6. Aggregation kinetics of SNCAΔ5 and SNCAΔ3Δ5 at lower protein concentrations (*n* ≥ 5). Averaged curves shown in **Fig. 3B** are shown as solid lines. Mean and SD values for t_{lag} and $t_{½}$ are reported.

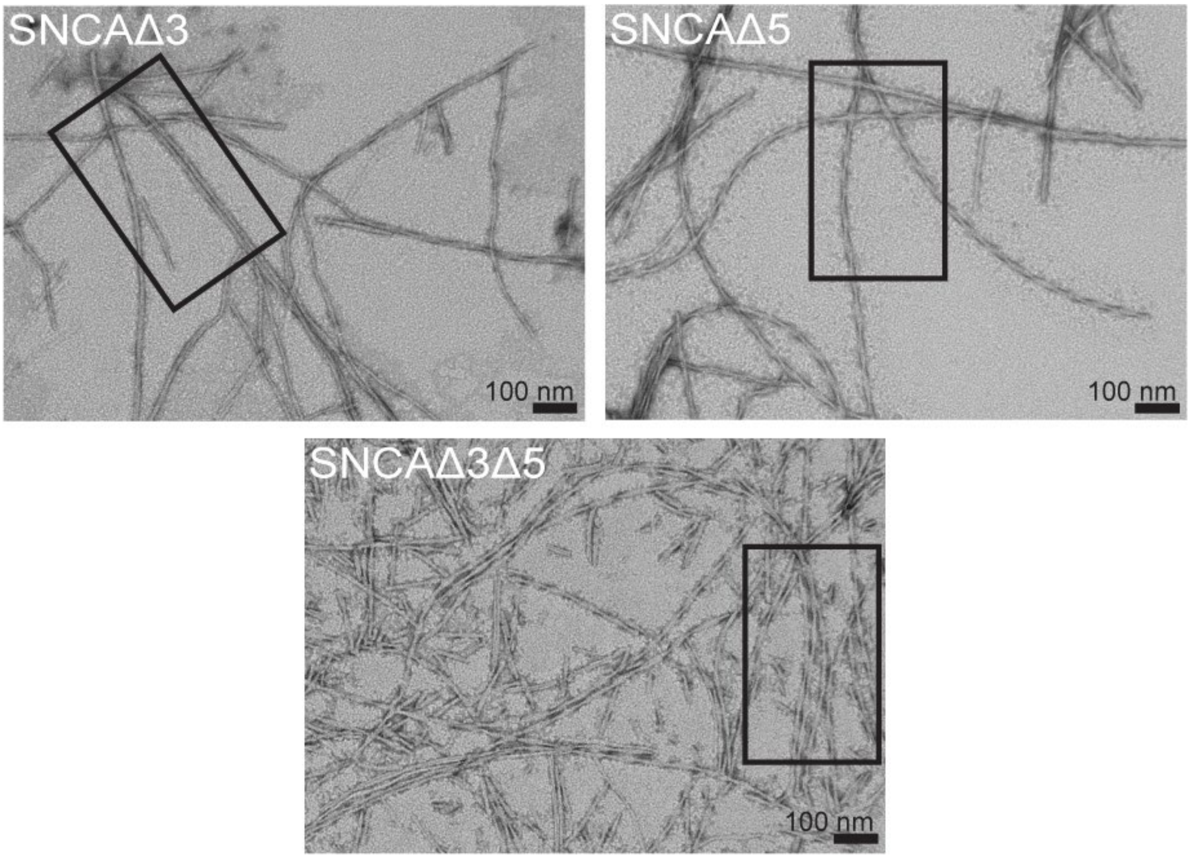

Figure S7. Full TEM images of fibrillar SNCA and spliced variants aggregated in the absence of beads. Fibrils were formed at 100 μM. Black box corresponds to field of view used in Fig. 3C.

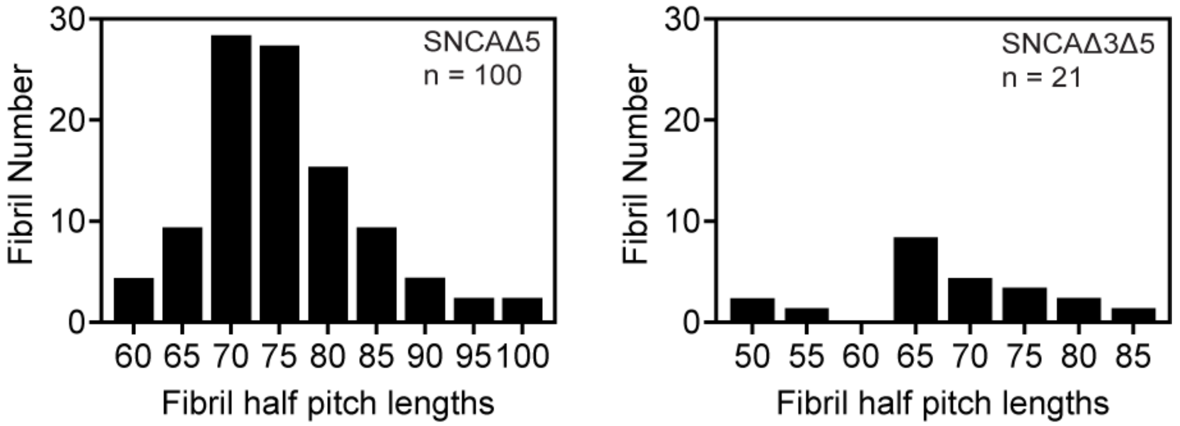

Figure S8. Histograms of measured fibril half-pitch lengths**.** Fibril half pitches of SNCAΔ5 (*n* = 100) and SNCAΔ3Δ5 (*n* = 21) are shown. Helical pitches were calculated using ImageJ.

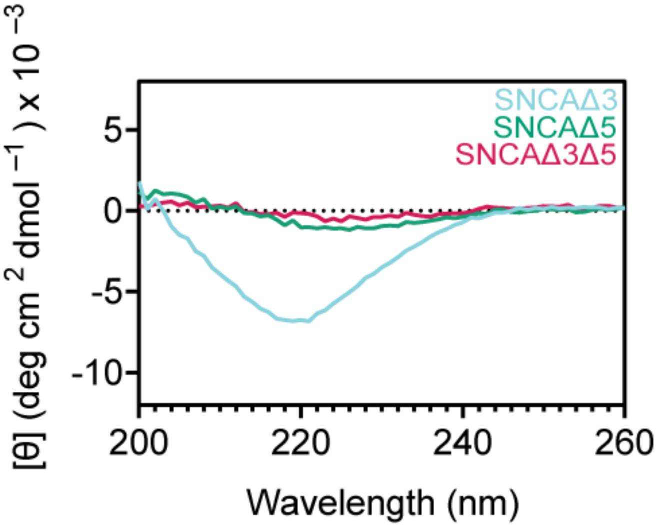

Figure S9. Comparison of CD spectra of spliced variants post-aggregation from low agitation conditions with no beads at pH 7.4.

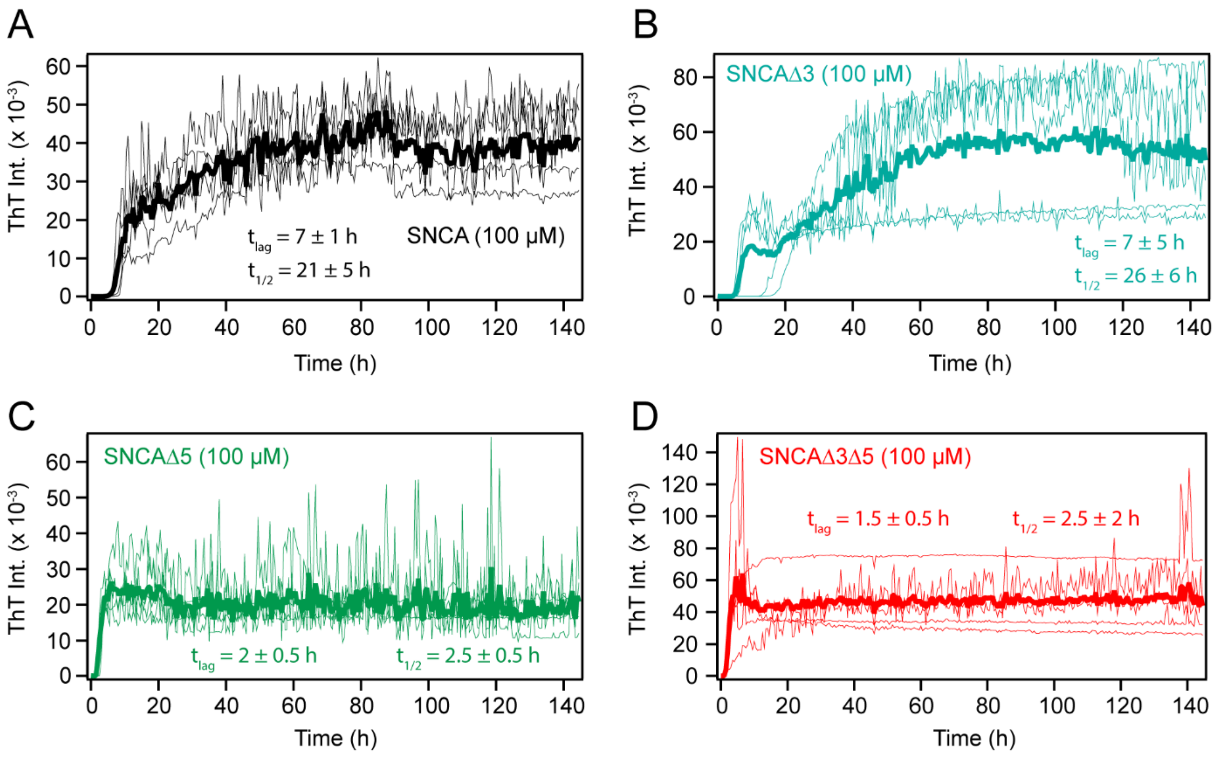

Figure S10. Aggregation kinetics of SNCA (A) and spliced variants (B–D) at 100 μM ($n \geq 4$, average shown as thick lines) at pH 5.0 (20 mM NaOAc, 140 mM NaCl, [ThT] = 20 mol%) under low agitation conditions in the absence of beads. Mean and SD values for t_{lag} and $t_{½}$ are reported.

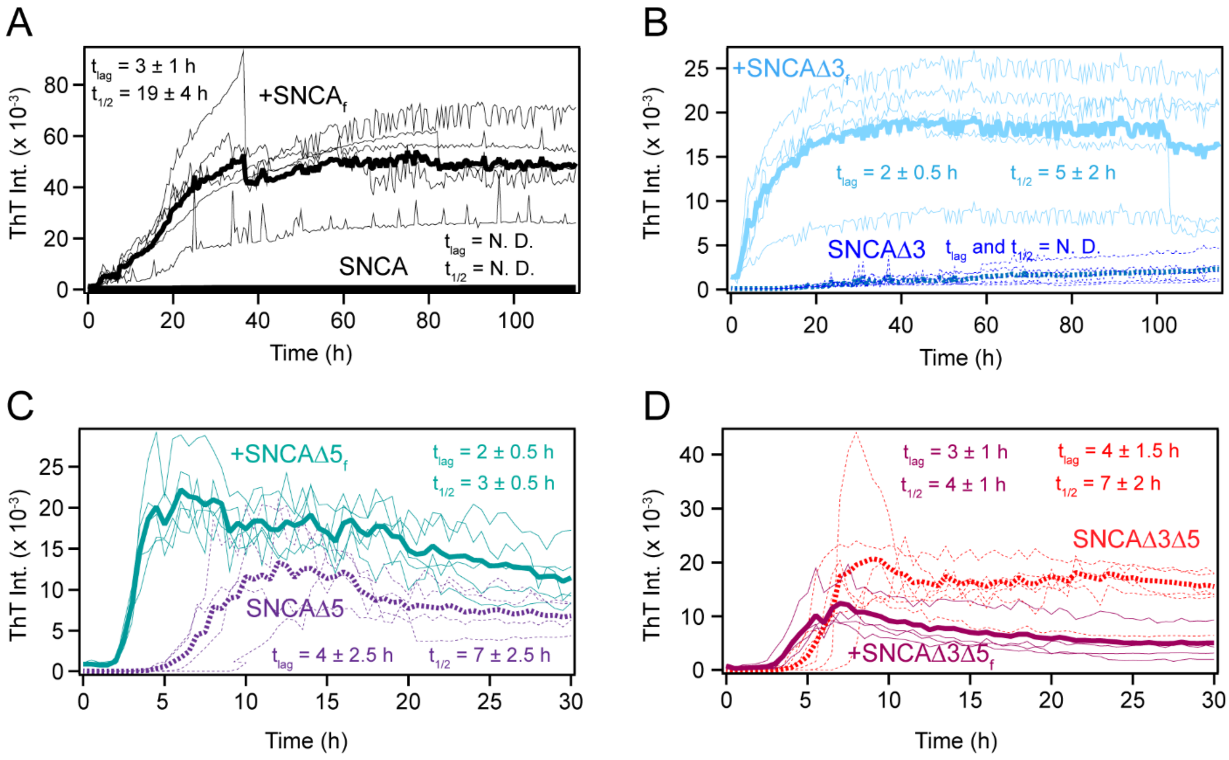

Figure S11. Self-seeding reactions of soluble SNCA (A) and alternatively spliced variants (B–D) in the presence of preformed 1.5 μM fibrils ($n \geq 5$, average shown as thick lines, in 20 mM NaPi, 140 mM NaCl, pH 7.4, [ThT] = 10 μM, [protein] = 30 μM). Subscript f denotes fibril. Mean and SD values for t_{lag} and $t_{½}$ are reported.

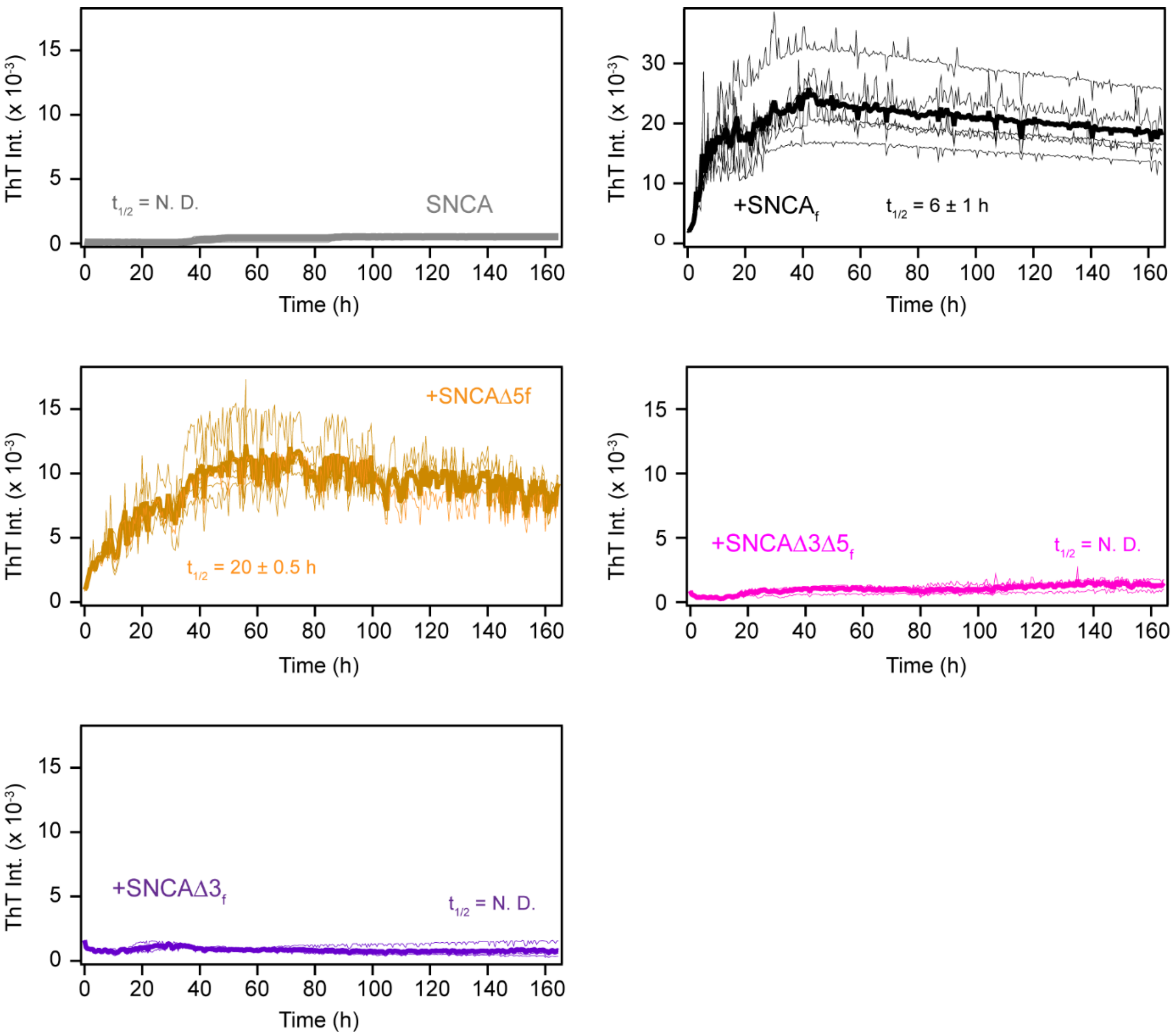

Figure S12. Cross-seeding reactions of soluble SNCA with alternatively spliced variant fibrils. Aggregation kinetics of SNCA (30 μM) in the presence of 1.5 μM preformed spliced variant fibrils (*n* = 5 in 20 mM NaPi, 140 mM NaCl, pH 7.4, [ThT] = 10 μM). Averaged curves shown in **Fig. 4A** are shown as solid lines. Mean values for $t_{½}$ are reported.

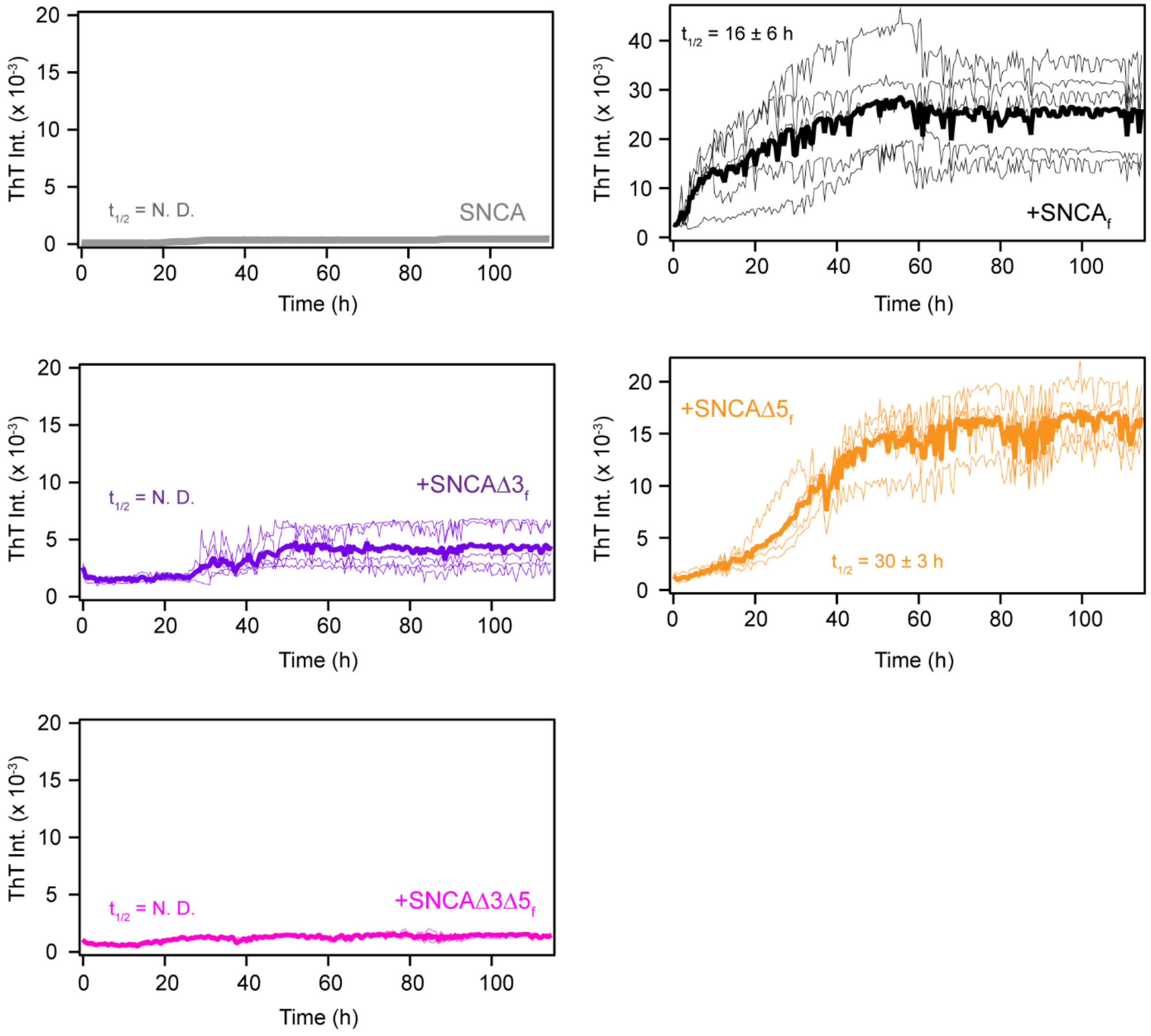

Figure S13. Aggregation kinetics of SNCA (30 μM) in the presence of 3 μM preformed spliced variant fibrils (n = 5, average shown in thick lines, in 20 mM NaPi, 140 mM NaCl, pH 7.4, [ThT] = 10 μM). Subscript f denotes fibril. Mean values for $t_{½}$ are reported.

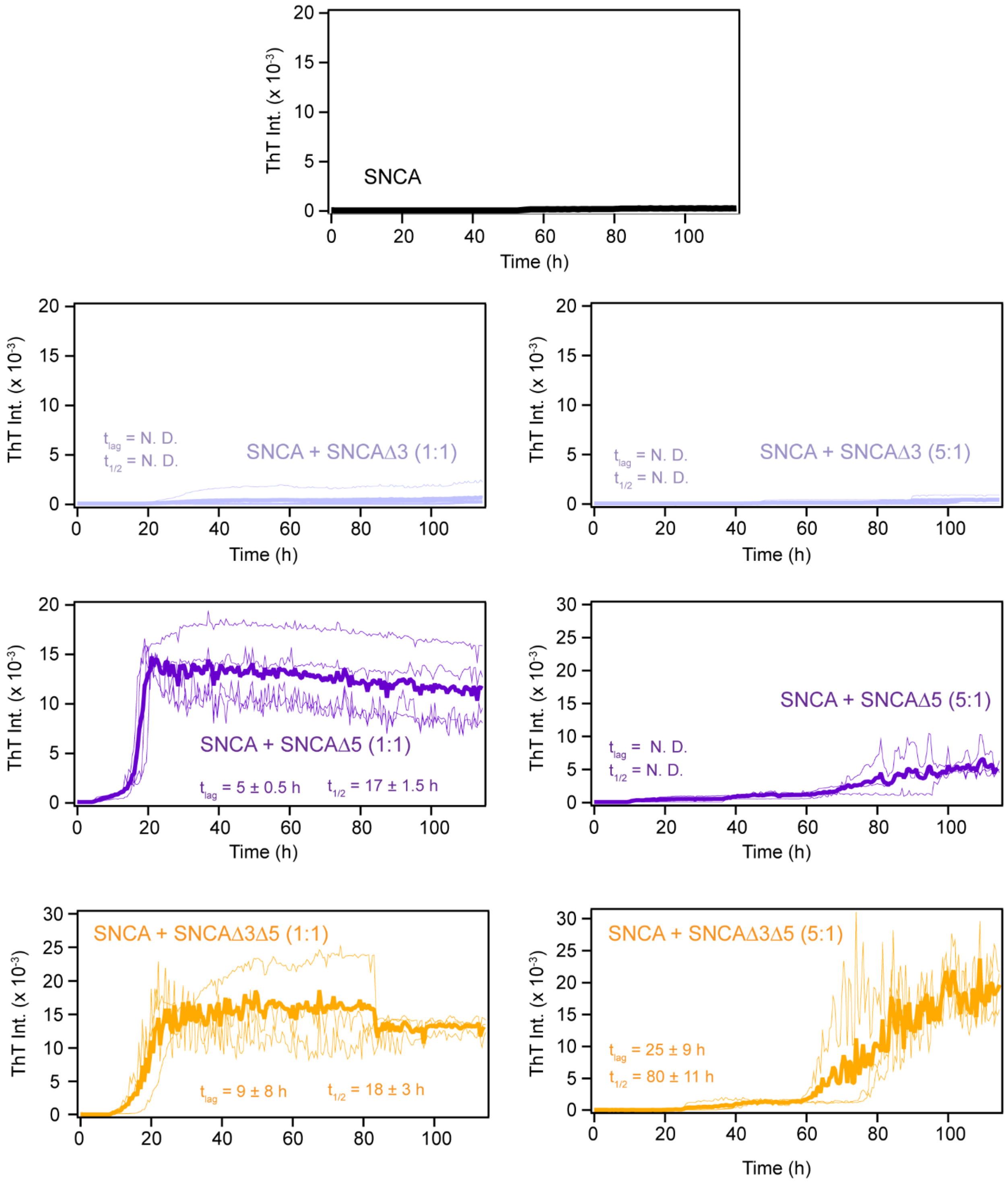

Figure S14. Co-mixing reactions of soluble SNCA with alternatively spliced variant monomer. Aggregation kinetics of SNCA (25 μM) in the presence of either 25 μM or 5 μM spliced variant monomer (indicated as 1:1 and 5:1, $n \geq 3$ in 20 mM NaPi, 140 mM NaCl, pH 7.4, [ThT] = 5 μM). Averaged curves shown in **Fig. 5A** and **B** are shown as solid lines. Mean and SD values for t_{lag} and $t_{½}$ are reported.
